# Boosting photocatalytic hydrogen evolution through efficient charge separation in NiSe_2_ nanooctahedra-TiO_2_ heterostructures

**DOI:** 10.1039/d6ra04767j

**Published:** 2026-08-11

**Authors:** Hicham Zalrhi, Mukul Sethi, Dirk Ziegenbalg, Lahoucine Atourki, Robert Güttel

**Affiliations:** a Institute of Chemical Engineering, Ulm University Albert-Einstein-Allee 11 Ulm 89081 Germany robert.guettel@uni-ulm.de; b MANAPSE Lab, Faculty of Science, Mohammed V University in Rabat Rabat 1014 Morocco; c Institute for Inorganic Chemistry I Albert-Einstein-Allee 11 Ulm 89081 Germany

## Abstract

The transition to clean energy has recently become the primary focus of researchers. Photocatalytic hydrogen evolution from aqueous sacrificial solutions using TiO_2_ as the photocatalyst has emerged as a viable approach. However, TiO_2_ is limited by its large band gap and rapid charge recombination. To overcome these limitations, we investigate the non-noble metal nickel diselenide (NiSe_2_), synthesized by a simple hydrothermal method, and a series of NiSe_2_/TiO_2_ (P25) composites prepared by a wet impregnation method. The intrinsic structural, morphological, optical, and electronic properties of the as-prepared NiSe_2_/TiO_2_ composites were investigated using a variety of characterization techniques. The introduction of nanooctahedral NiSe_2_ led to a 258-fold enhancement in hydrogen evolution compared to pristine TiO_2_ under UV irradiation. The optimized catalyst exhibited a hydrogen evolution rate of ≈129.4 µmol h^−1^ and an apparent quantum efficiency (AQE) of ≈4% at 365 nm. This enhancement is attributed to improved charge separation and transfer arising from the morphology, high conductivity, and promising catalytic activity of NiSe_2_, enabling efficient hydrogen production. Furthermore, the comparison of sacrificial agents shows that for methanol, CO_2_ was the only detected carbon-containing gaseous product under the applied conditions, whereas glycerol reforming resulted in the formation of both CO and CO_2_. During glycerol reforming, post-reaction ^1^H NMR analysis did not reveal major detectable liquid-phase intermediates within the sensitivity limits of the measurement. To further investigate the effect of NiSe_2_ on the photocatalytic hydrogen evolution of TiO_2_, a reaction mechanism is proposed. This study demonstrates the potential of nanooctahedral NiSe_2_ as a cost-effective, stable, and efficient non-noble metal co-catalyst for hydrogen evolution.

## Abstract 2

NiSe_2_ nanooctahedral cocatalysts significantly enhance TiO_2_ photocatalytic hydrogen production by promoting charge separation and surpassing photogenerated charge recombination.

## Graphical abstract

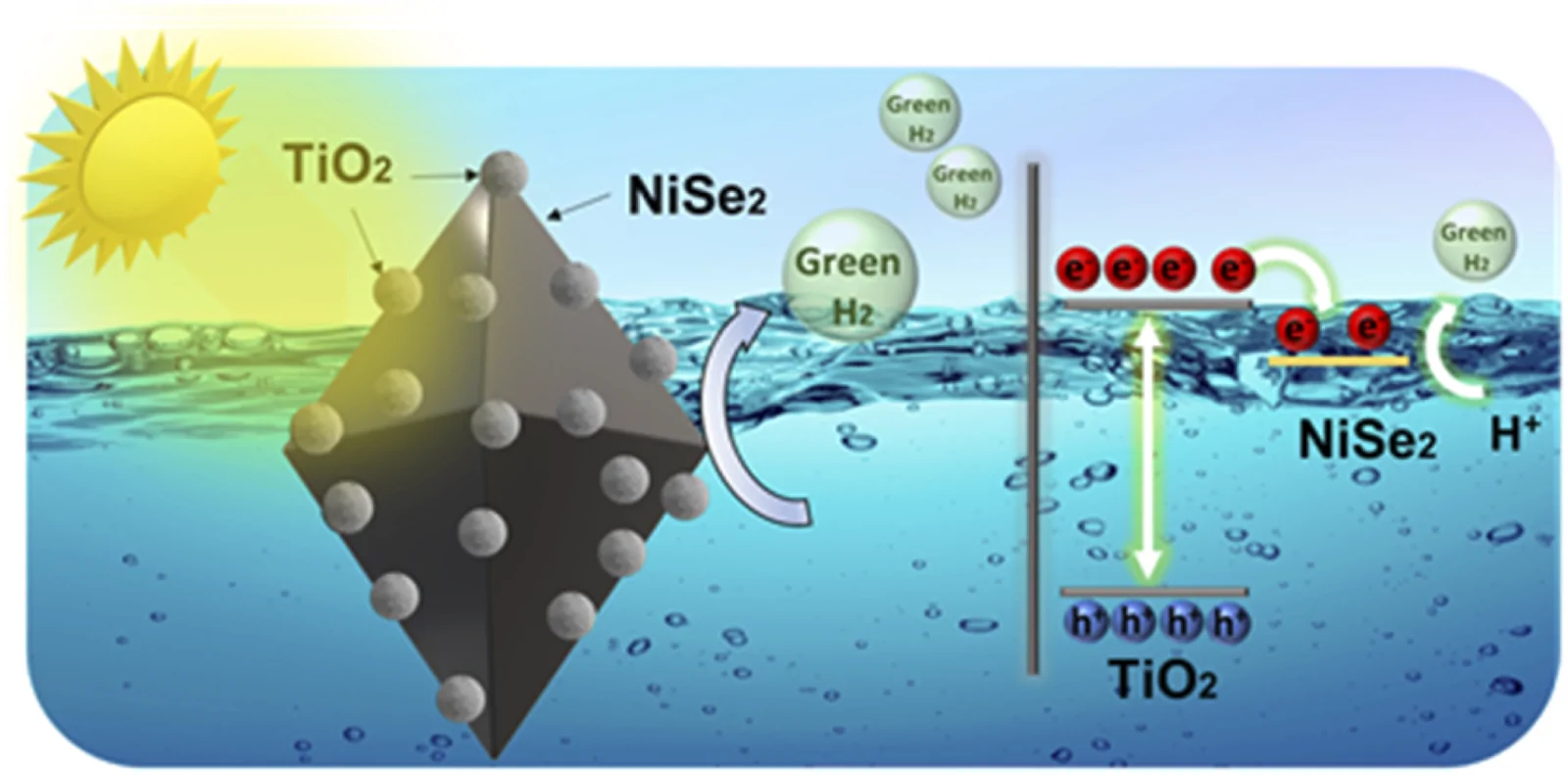

## 1 Introduction

Hydrogen has recently received much attention from academia and industry due to its diverse applications, including power systems for hydrogen production, re-electrification, and storage.^1^ Among the different types of hydrogen, green hydrogen has emerged as a promising alternative to fossil fuels with no emission of greenhouse gases, offering high energy density (*ca.* 120 MJ kg^−1^) and lower volumetric energy density (*ca.* 8 MJ L^−1^).^2–4^ The growing interest has led to significant advances in developing new materials and architectures toward achieving effective materials that can convert light into clean, valuable energy. To be named green hydrogen, the energy used to produce hydrogen must be clean, environmentally friendly, and based on a renewable source. Various processes are available, including photocatalytic and photoelectrocatalytic hydrogen production, which have proven their effectiveness in producing pure hydrogen.

Photocatalytic hydrogen evolution is a promising method for hydrogen generation.^5–7^ Notably, alcohol photoreforming has emerged as an efficient strategy because alcohols readily consume photogenerated holes, suppress electron–hole recombination, and enhance hydrogen evolution compared with conventional overall water splitting.^8,9^ Among various sacrificial agents, methanol is one of the most widely used owing to its low oxidation potential, rapid oxidation kinetics, complete miscibility with water, and well-established reaction pathway. Consequently, alcohol dehydrogenation by photocatalysis demonstrates its effectiveness and environmental compatibility. Compared to all available photocatalysts, including TiO_2_,^10,11^ CdS,^12–14^ BiVO_4_,^15^ g-C_3_N_4_,^16,17^ CuFeO_2_,^18,19^ TiO_2_ is considered one of the most promising due to its properties. First, it is made of cheap, abundant, and non-toxic elements, which are important characteristics for large-scale applications. Additionally, it is stable in aqueous conditions, making it a suitable choice for photocatalytic hydrogen production. Nevertheless, TiO_2_ suffers from various limitations, including its wide band gap, which restricts absorption to UV light, and rapid recombination of photogenerated charge carriers, which leads to low photocatalytic performance.

Meanwhile, various strategies can overcome these limitations, including elemental doping,^20^ co-catalyst loading,^21–23^ and heterojunction formation with other semiconductors. Co-catalysts demonstrated their effectiveness towards a dramatic improvement in hydrogen production. Noble metal cocatalysts were found to have the highest activity. However, although there are many reports on the advantages of noble metals,^24,25^ the cost and availability of these materials pose major limitations. This pushes researchers to look for non-noble metals that can lead to nearly similar performance enhancement.

Ni-based chalcogenide materials, such as NiSe_2_, have been considered promising co-catalysts, with their high conductivity, catalytic activity, stability, and abundance making them top alternatives to noble metal co-catalysts. NiSe_2_ has been used extensively in many applications, including energy storage,^26^ electrocatalysis, and high-temperature superconductors,^27^ and recently the focus has turned to photocatalytic hydrogen production.^28^ Moreover, the morphology of NiSe_2_ can significantly affect the material's properties. For instance, NiSe_2_ and doped NiSe_2_ nanosheets were prepared and found to improve the activity of ref. 29 and 30. NiSe_2_ nanoparticles loaded on CdS nanorods for hydrogen production were found to increase hydrogen generation by 2.7 and 2.6 times, respectively, compared to pristine CdS.^31,32^ The octahedral morphology of NiSe_2_ has attracted great interest in recent years, as many researchers have investigated its effectiveness for batteries and as an electrode for electrocatalytic water splitting.^33–35^ Khan *et al.*^36^ successfully grew NiSe_2_ nanooctahedra on NiTiO_3_, demonstrating improved efficiency and selectivity for photocatalytic CO_2_ reduction.

Overall, NiSe_2_ nanooctahedra have attracted considerable attention for energy storage and CO_2_ photoreduction because of their high electrical conductivity, well-defined crystal facets, and excellent catalytic activity; however, despite their advantages, their application in photocatalytic hydrogen evolution remains largely unexplored. Motivated by this gap, the present work investigates morphology-controlled NiSe_2_ nanooctahedra as a noble metal-free cocatalyst for TiO_2_ toward photocatalytic hydrogen evolution. In addition to evaluating photocatalytic activity, a systematic investigation of cocatalyst loading, catalyst amount, sacrificial-agent type and concentration, photoelectrochemical behavior, apparent quantum efficiency, and catalyst stability was performed. Under the optimized conditions, the NiSe_2_/TiO_2_ heterostructure achieved a hydrogen evolution rate of 129.4 µmol h^−1^ (2588 µmol h^−1^ g^−1^), corresponding to a 258-fold enhancement over pristine TiO_2_.

## 2 Experimental section

### 2.1 NiSe_2_ synthesis

**Fig. 1:**
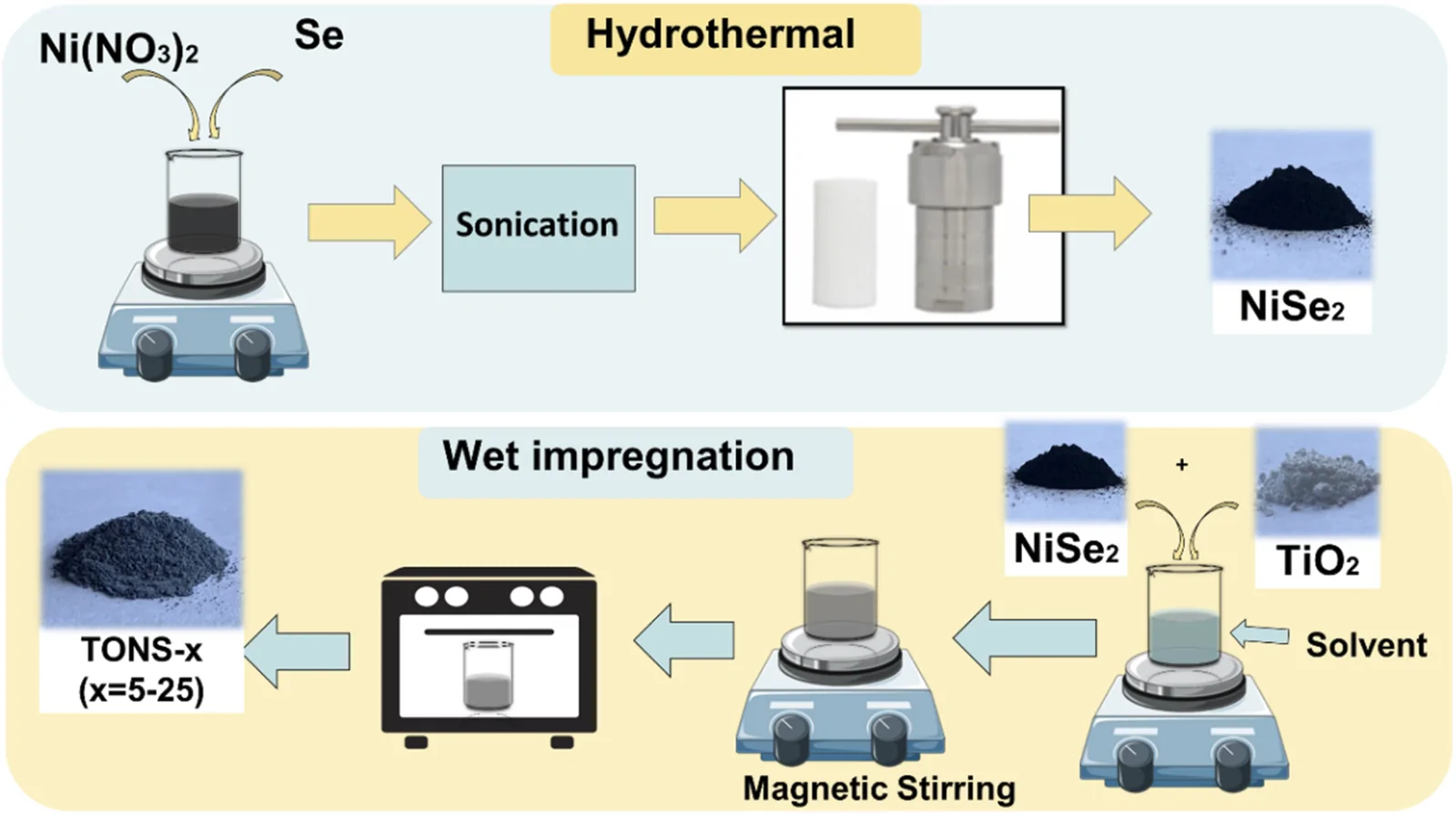
Schematic illustration of the synthesis of NiSe_2_ nanooctahedra and preparation of NiSe_2_/TiO_2_ heterostructures *via* hydrothermal and wet impregnation methods.

NiSe_2_ materials with a positive octahedral structure were synthesized by a simple hydrothermal method. First, 0.52 g of Se powder was added to a beaker containing 5 mL of hydrazine hydrate, followed by sonication for 30 minutes. A second solution was prepared by dissolving nickel nitrate in deionized (DI) water and stirring it magnetically for 15 minutes. Solution 2 was then added to Solution 1, and the mixture was sonicated for an additional 30 minutes. The resulting mixture was transferred into a 20 mL stainless steel autoclave and heated at 180 °C for 24 hours. After the reaction, the autoclave was allowed to cool naturally to room temperature (Fig. 1). The black precipitate obtained was collected by centrifugation and washed several times with anhydrous ethanol. The product was then dispersed again in anhydrous ethanol and finally dried in an oven at 65 °C for 24 h to obtain the desired powder sample.

### 2.2 Synthesis of octahedral NiSe_2_ on TiO_2_

To prepare NiSe_2_-loaded TiO_2_, the desired amount of TiO_2_ (P25) was dispersed in 10 mL of a water/ethanol mixture (1 : 1 v/v) and magnetically stirred until a homogeneous white suspension was obtained. NiSe_2_ was then slowly added to the mixture, causing the color to change to dark gray gradually, as shown in Fig. 1. The suspension was maintained under constant magnetic stirring at 90 °C until it was completely dried. The resulting solid was subsequently placed in an oven at 70 °C for 24 h. The final powders were referenced as TONS-*x*, where *x* represents the weight percentage of NiSe_2_ (5–25 wt% of the total mass).

### 2.3 Material characterization

XRD measurements were performed in reflection mode on an X-ray diffractometer (X'Pert Pro, Malvern Panalytical) with CuK_α_. The crystal structure was identified and refined with the Rietveld method using the “FullProf” software. SEM images were obtained using a Zeiss Sigma VP scanning electron microscope operated at an accelerating voltage of 2 kV, equipped with an in-lens detector. For transmission electron microscopy, the sample was prepared on TEM grids coated with lacey carbon films. High-resolution TEM (HRTEM), high-angle annular dark-field scanning TEM (HAADF-STEM) imaging, and energy dispersive X-ray (EDX) mapping were acquired on a 24 Thermo Fisher Talos 200X equipped with a SuperX four-quadrant EDX detector. X-ray photoelectron spectroscopy (XPS) measurements were performed using a commercial Physical Electronics PHI 5800 ESCA system equipped with Kα X-ray source. UV-Vis diffuse reflectance spectra (DRS) were recorded using a UV-Vis-NIR spectrophotometer (Lambda 1050, PerkinElmer) with BaSO_4_ as the reference standard. Emission spectroscopy was performed on a JASCO FP-8500 spectrofluorometer. Quartz glass cuvettes (*d* = 10.0 mm) were used for optical measurements. The textural properties of the catalysts were evaluated by nitrogen physisorption measurements using a 3Flex analyzer (Micromeritics). Prior to analysis, the samples were degassed at 200 °C under reduced pressure (1.33 mbar). Adsorption–desorption isotherms were recorded at 77 K over a relative pressure (*P*/*P*_0_) range of 0.01–0.99. The specific surface areas were determined using the Brunauer–Emmett–Teller (BET) method. Elemental leaching after the photocatalytic reaction was investigated by inductively coupled plasma optical emission spectroscopy (ICP-OES) using a Spectro Arcos FHS12 spectrometer (Spectro Analytical Instruments). During sample preparation, the sample was diluted to a factor of 5 to raise the volume of the solution and for matrix matching. Nuclear magnetic resonance (NMR) measurements were performed using a Bruker AVANCE NEO 400 spectrometer equipped with a Bruker iProbe TBO BBFO/F/H probehead with *Z*-gradient. All measurements were conducted under standard temperature conditions.

### 2.4 Hydrogen evolution experiment

Photocatalytic hydrogen generation was carried out in a modular photoreactor equipped with a 365 nm UV LED light source and a 100 mL borosilicate reactor. The modular photoreactor was laboratory-fabricated using 3D printing, and the experimental setup and irradiation conditions were adapted from previously reported procedures.^37,38^ The incident photon flux determined by ferrioxalate actinometry was approximately 1.8 × 10^−6^ mol s^−1^ under the applied irradiation conditions. To measure the hydrogen evolution, 50 mg of the catalyst was dispersed in 100 mL of deionized water containing 25 mL of methanol. The suspension was purged with argon at a flow rate of 20 mL min^−1^ using a mass flow controller to remove oxygen under constant stirring at 500 rpm. The evolved hydrogen was detected at regular intervals (every 11 min) using an online gas chromatography system (Thermo Scientific TRACE 1310 GC) equipped with a thermal conductivity detector (TCD). The experiments were repeated at least two times to confirm reproducibility. The experimental error associated with photocatalytic hydrogen production was estimated to be approximately 2–5%. The experimental setup and irradiation conditions are illustrated in Fig. S1.

### 2.5 Photoelectrochemical experiment

The photoelectrochemical (PEC) properties of the catalysts were investigated using a standard three-electrode electrochemical workstation (Gamry interface 1010 potentiostat). Electrochemical impedance spectroscopy (EIS) and transient photocurrent responses were measured. The PEC measurement was conducted using the same modular photoreactor as explained in the photocatalytic measurement (Fig. S2). The test system consisted of a three-electrode system, with 0.5 M Na_2_SO_4_ solution as an electrolyte, and a platinum electrode as the counter electrode and AgCl/Ag as the reference electrode. To prepare the working electrodes, the drop-casting deposition method was used. First, the FTO substrate was cleaned in an ultrasonic bath for 20 minutes. Then, 10 mg of the catalyst was weighed and dispersed in a solution containing 1 mL of ethanol, and 10 µL of Nafion was added to stabilize the suspension. Subsequently, 80 µL of the suspension was drop-cast onto the FTO substrate and left to dry overnight.

## 3 Results and discussion

**Fig. 2:**
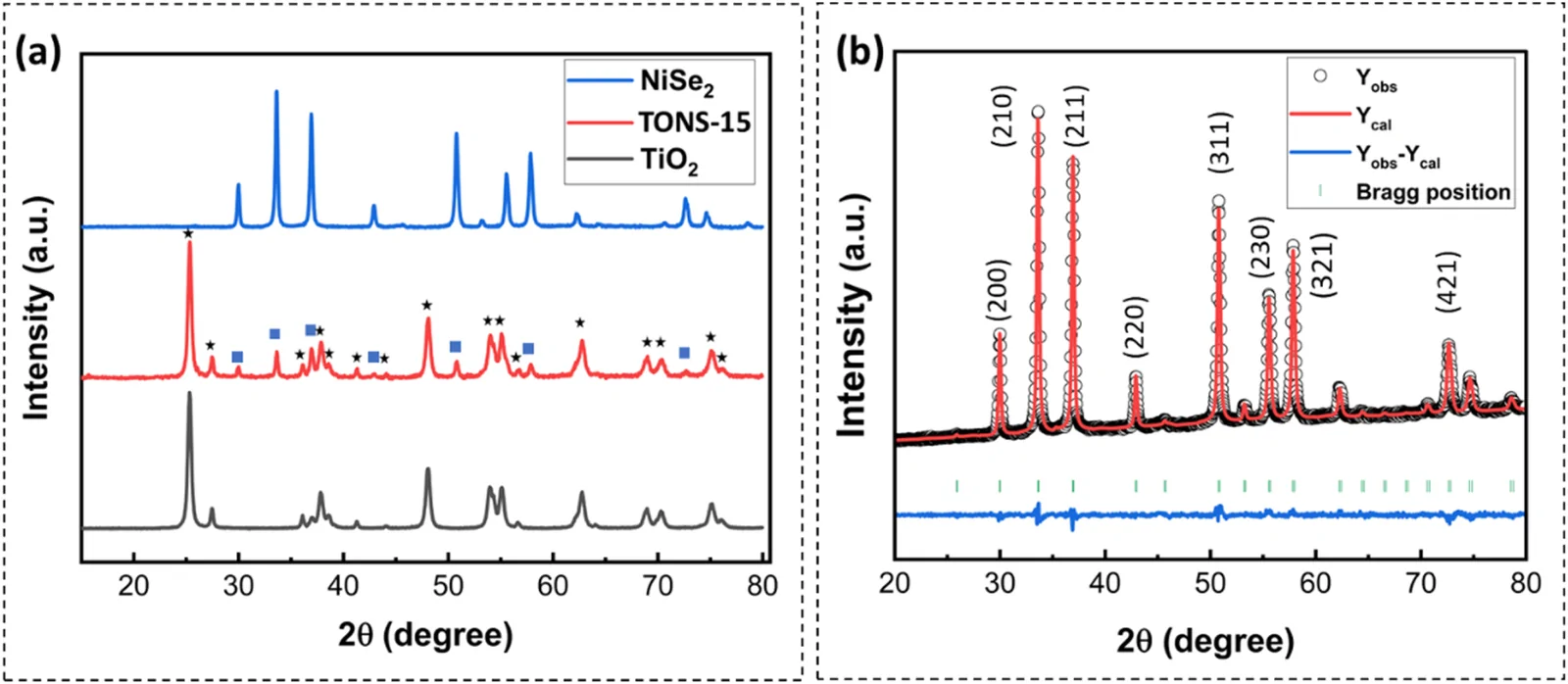
(a) The XRD pattern of TiO_2_, NiSe_2_, and their composite, (b) the Rietveld refinement of the as-prepared NiSe_2_.

The powder XRD patterns of the pure NiSe_2_, pure TiO_2_, and the synthesized NiSe_2_/TiO_2_ heterostructure are shown in Fig. 2a. First, two intense diffraction peaks of TiO_2_ at 2*θ* = 25.28° and 27.446° can be seen, which correspond to the (101) and (110) lattice planes, respectively. This is attributed to the coexistence of anatase and rutile phases in P25. The XRD pattern of the nano-octahedral NiSe_2_ sample was also investigated. The Rietveld refinement was conducted to further confirm the purity and determine the lattice parameters of NiSe_2_ (Fig. 2b). The three strong peaks at 2*θ* = 33.6°, 36.9°, and 50° correspond to the (210), (211), and (311) crystal planes of the NiSe_2_ standard card, with crystal plane spacings of *d* = 0.27 nm, 0.24 nm, and 0.18 nm, respectively. The refined theoretical lines fit well with the experimental profiles, as confirmed by the goodness of fit (*χ*^2^) of 1.18. The Rietveld analysis verifies that the refined parameters correspond to the Pbnm space group, associated with an orthorhombic structure. No impurity peaks are observed, indicating high purity and crystallinity. The TONS-15 sample shows a mixture of NiSe_2_ and TiO_2_ peaks, with stronger TiO_2_ peaks compared to NiSe_2_, consistent with the composition ratio (Fig. S4a). Additionally, the lattice parameters resulted from the Rietveld refinement of NiSe_2_ (Fig. 2b), TiO_2_ (Fig. S4a), and TONS-15 (Fig. S4b) demonstrate that there is no change in crystal structure before and after introducing NiSe_2_ on TiO_2_, as shown in Table S1, indicating that octahedral NiSe_2_ plays only the role of a co-catalyst and does not affect the structural properties of TiO_2_.

**Fig. 3:**
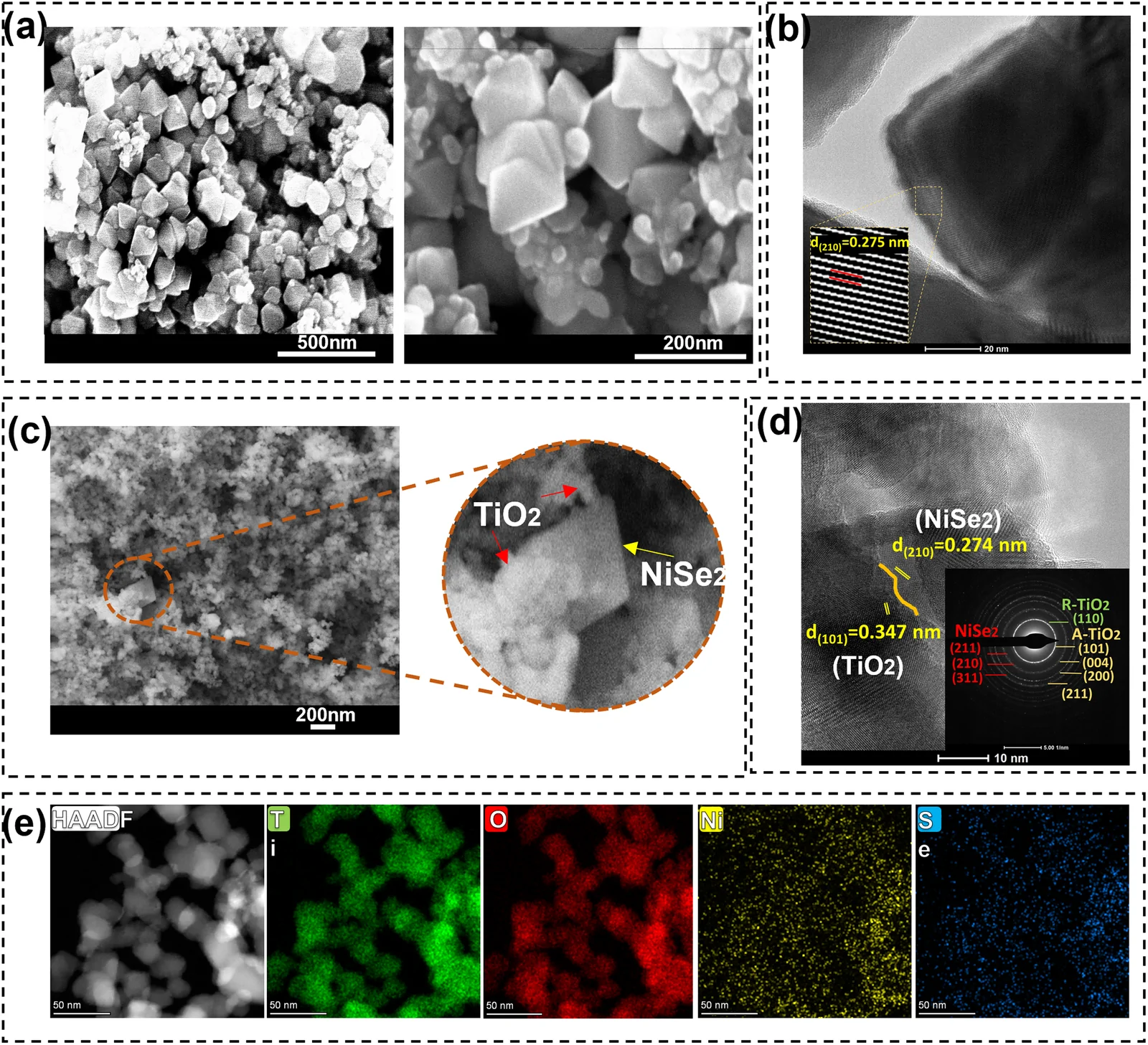
Morphological characterization of the NiSe_2_ nanooctahedra and NiSe_2_/TiO_2_ heterostructure: (a) SEM images at different magnifications, (b) TEM image of NiSe_2_ nanooctahedra, (c) SEM image of the NiSe_2_/TiO_2_ heterostructure with a zoomed-in view highlighting the interfacial contact between TiO_2_ and NiSe_2_, (d) HRTEM image of the NiSe_2_/TiO_2_ interface with the corresponding SAED pattern shown in the inset, (e) HAADF elemental mapping of TONS-15.

Fig. 3 illustrates the nanostructure and morphology of pure NiSe_2_ octahedra and the NiSe_2_/TiO_2_ mixture characterized by SEM and TEM analyses. The SEM analysis, as shown in Fig. 3a, presents pristine NiSe_2_ at different magnifications, clearly demonstrating a typical nano-octahedral morphology with an average size of around 150 nm. The geometric nano-octahedral structure is further confirmed by the TEM images, which are consistent with the SEM observations. The HRTEM image (Fig. 3b) of the NiSe_2_ nano-octahedra exhibits clear lattice fringes with a spacing of 0.275 nm, which can be indexed to the (210) plane of NiSe_2_, commonly observed in NiSe_2_ nano-octahedra.^39,40^Fig. 3c shows the SEM image of the TiO_2_/NiSe_2_ (15 wt% NiSe_2_) composite, indicating that TiO_2_ nanoparticles cover the surface of NiSe_2_, which is further confirmed by the particle size difference between NiSe_2_ and TiO_2_. The intimate contact between the two components is clearly observed in Fig. 3d, showing a *d*-spacing of 0.34 nm corresponding to anatase TiO_2_, as also observed in the SAED patterns along with NiSe_2_ and rutile TiO_2_. In addition, a lattice spacing of 0.27 nm corresponding to NiSe_2_ is observed, consistent with the (210) plane of the NiSe_2_ single crystal. The elemental composition of the NiSe_2_ and NiSe_2_/TiO_2_ composites was further confirmed by EDX analysis, as shown in Fig. S5 and S6, verifying the successful incorporation of NiSe_2_ onto the TiO_2_ surface. Moreover, HAADF and elemental mapping confirm the uniform dispersion of NiSe_2_ on the TiO_2_ surface and the formation of a well-defined heterostructure with intimate interfacial contact (Fig. 3e), which is favorable for enhanced interfacial charge transfer and improved photocatalytic hydrogen evolution.

**Fig. 4:**
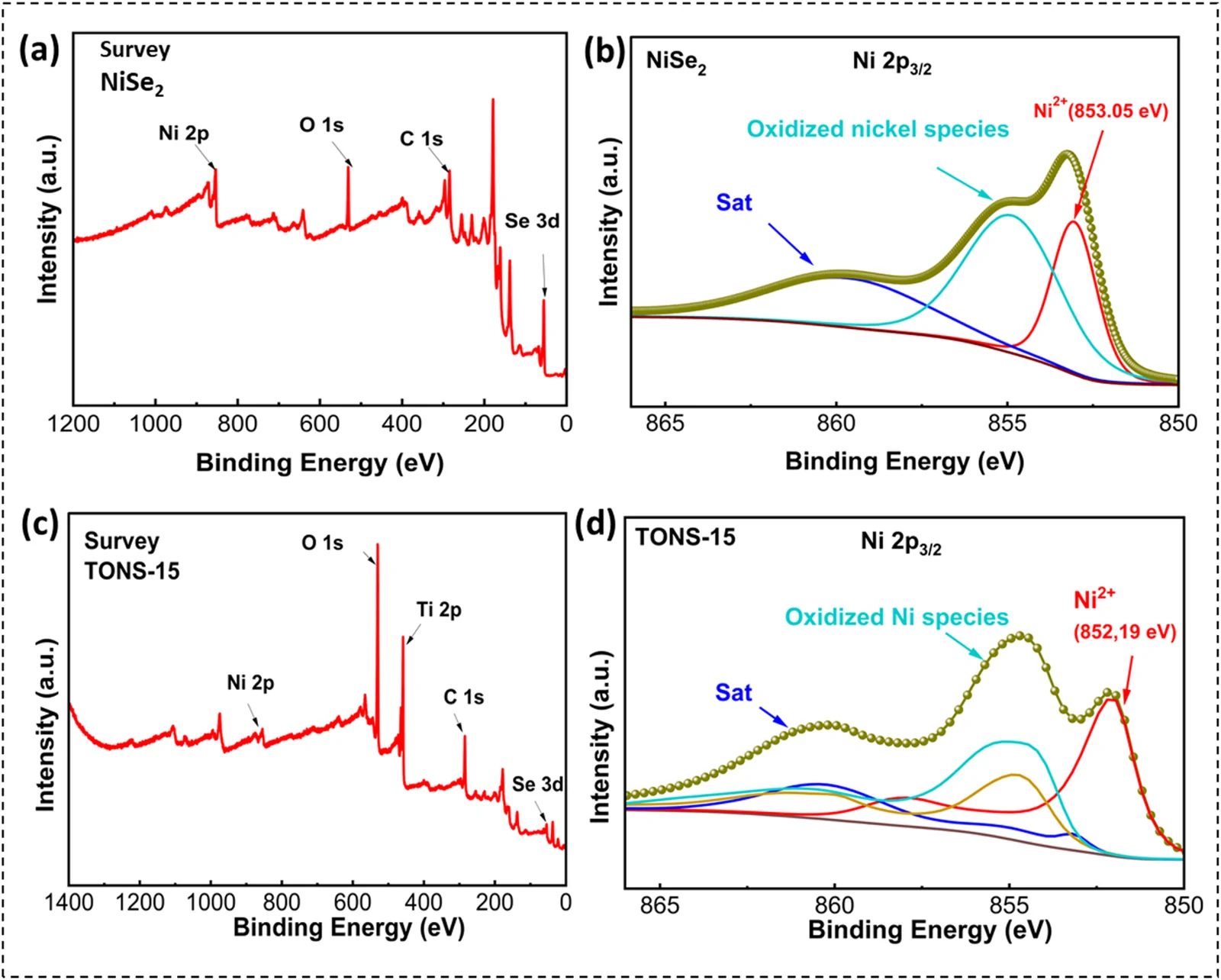
(a) Survey spectra and (b) high-resolution spectra of Ni 2p for NiSe_2_. (c) Survey spectra and (d) high-resolution spectra of Ni 2p for TONS-15.

To better understand the chemical states of surface atoms in the as-prepared NiSe_2_ and their impact on TiO_2_, XPS measurements were carried out. The survey spectra of the NiSe_2_ nanooctahedrons reveal distinct photoelectron signals from Ni and Se, along with O arising from surface oxidation (Fig. 4a). The corresponding high-resolution Ni 2p and Se 3d spectra of the NiSe_2_ nanooctahedrons are shown in Fig. 4b and S7a, respectively. The deconvoluted Ni 2p spectrum of pure NiSe_2_ shows a prominent peak at 853.05 eV, corresponding to Ni 2p_3/2_ electrons of Ni^2+^. The peak at 855 eV is related to the oxidation state of Ni on the surface. A satellite peak at a binding energy of 860.1 eV was also detected. In addition, the Se 3d spectrum, as shown in Fig. S7a, exhibits two peaks at 54.91 eV and 56.26 eV, corresponding to Se 3d_5/2_ and Se 3d_3/2,_ respectively, while the presence of a peak at 59.1 eV is due to surface oxidation. The survey spectra of TONS-15 exhibit photoelectron signals from Ti, O, Ni, and Se from NiSe_2_, with a dominant presence of TiO_2_ (Fig. 4c). However, the Ni 2p spectrum after loading NiSe_2_ nanooctahedra on TiO_2_ exhibits peaks at binding energies of 852.19 eV corresponding to the Ni 2p_3/2_. Compared with pure NiSe_2_, TONS-15 shows a shift in the characteristic peaks of Ni by 0.86 eV to lower binding energy. This clear Ni 2p peak shift toward lower binding energy suggests electronic interaction and possible charge transfer between TiO_2_ and NiSe_2_. The presence of Se is demonstrated in Fig. S7b; the peaks at 54.19 and 54.95 eV correspond to Se 3d_5/2_ and Se 3d_3/2,_ respectively, while the other two peaks might arise from surface oxidations, as demonstrated by various findings. Additionally, the presence of TiO_2_ was confirmed through the intense peaks of Ti 2p and O 1s, as shown in Fig. S7c and d.

**Fig. 5:**
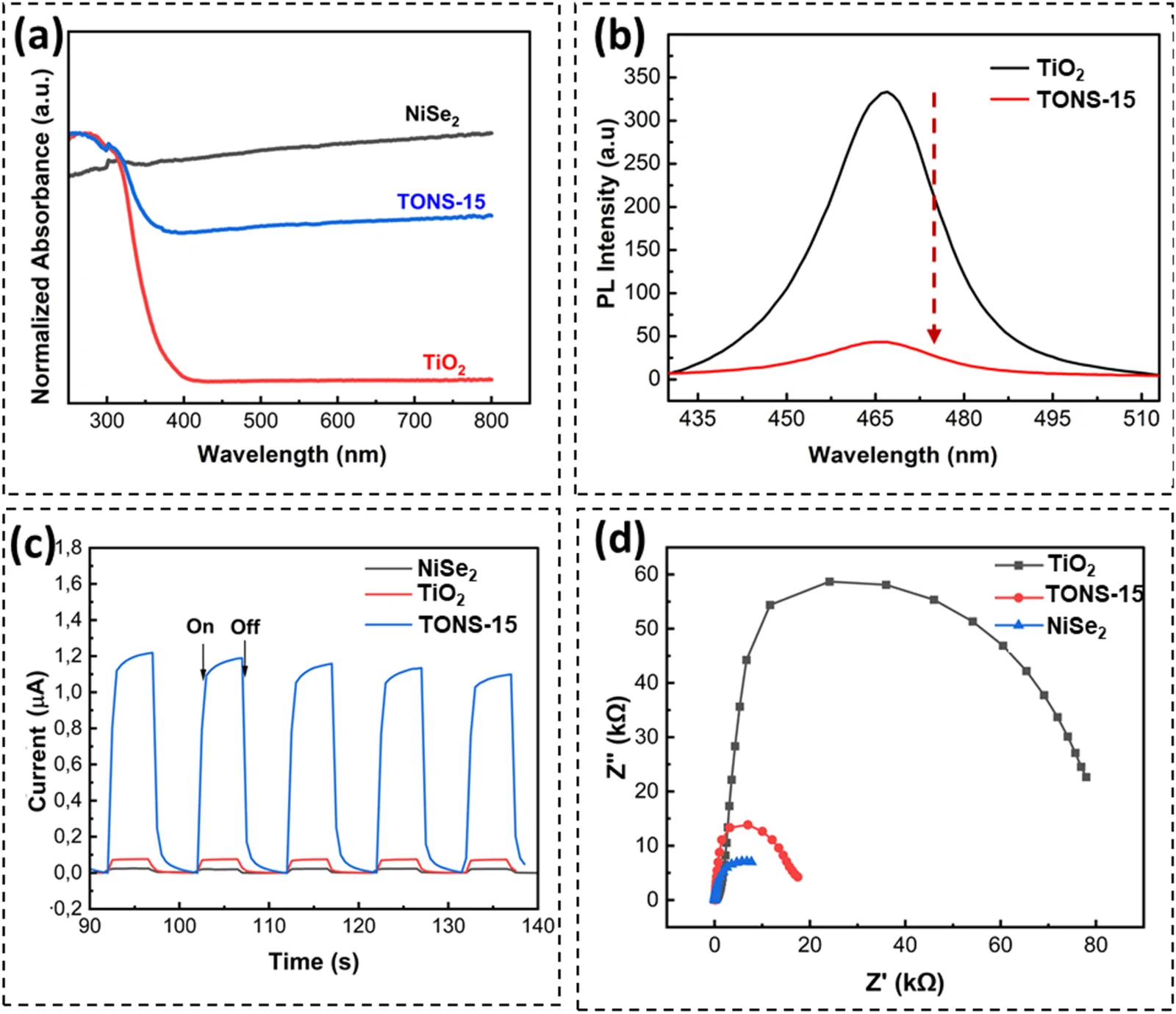
(a) UV-Vis diffuse reflectance spectra of TiO_2_, NiSe_2_, and TONS-15 samples. (b) PL spectra of TiO_2_ and TONS-15. (c) Transient photocurrent responses of TiO_2_, NiSe_2_, and TONS-15 under intermittent light irradiation. (d) Electrochemical impedance spectroscopy (EIS) Nyquist plots of TiO_2_, NiSe_2_, and TONS-15.

The optical absorption, photocurrent response, and electrochemical impedance measurements were used to further evaluate the enhancement in the NiSe_2_/TiO_2_ contact and photoresponse. First, the absorbance spectra of pure NiSe_2_, TiO_2_, and TONS-15 were obtained in the range of 250–800 nm (Fig. 5a). The pure NiSe_2_ nanooctahedra show relatively constant absorption over the selected wavelength range, indicating the conductive nature of NiSe_2_, while TiO_2_ absorbs mainly in the UV region. The absorption spectrum of TONS-15 shows nearly identical absorption characteristics to pristine TiO_2,_ with only slight extension toward the visible light. Tauc plots show comparable band gap energies of 3.24 and 3.20 of TiO_2_ and TONS-15, respectively (Fig. S8), which demonstrates that NiSe_2_ forms a heterostructure without significantly changing the absorption properties of TiO_2._ It is worth noting that the observed unchanged optical and structural properties, as supported by the nearly constant lattice parameters observed in the XRD and the Rietveld refinement, together with the binding-energy shifts in Ni 2P and Se 3d spectra, suggest that the material undergoes an electronic redistribution at the NiSe_2_/TiO_2_ interface rather than from bulk lattice modification. This provides further evidence for strong interfacial coupling between the two components. Fig. 5b shows the PL spectra of TiO_2_ and TONS-15 samples. TiO_2_ exhibits the highest emission peak, which indicates a high radiative recombination rate. After loading NiSe_2_ on TiO_2_, a marked reduction in PL intensity was observed. This quenching suggests a reduction of radiative recombination and is consistent with improved charge separation in the heterostructure.^41,42^ The photocurrent was evaluated by switching the light on and off every 5 s (Fig. 5c). NiSe_2_ was found to have the lowest generated photocurrent of 0.025 µA, which aligns with its metallic nature. On the other hand, TiO_2_ exhibits a 0.076 µA; this low photogenerated current is mainly due to the high recombination rate of charge carriers, which limits its performance. Using NiSe_2_ as a co-catalyst on TiO_2_ significantly enhances charge transfer and improves photoresponse, reaching 1.2 µA, which is 15.8 times higher than pristine TiO_2_. The EIS was used to further investigate charge separation and transfer efficiency in the three samples. TiO_2_ shows a higher charge-transfer resistance between the sample and the electrolyte, while NiSe_2_ exhibits high conductivity. TiO_2_ loaded with NiSe_2_ shows a dramatic decrease in charge-transfer resistance, while NiSe_2_ likely acts as an effective electron sink, which could contribute to reduced recombination of photogenerated electron–hole pairs, which is a major limitation of TiO_2_ photocatalysts.

The textural properties of the samples were investigated by N_2_ adsorption–desorption measurements (Fig. S9) and summarized in Table S2. Pristine NiSe_2_ exhibits a very low BET surface area of 3.63 m^2^ g^−1^, which is consistent with the large particle size observed in SEM and TEM images. In contrast, commercial P25 TiO_2_ shows a significantly higher surface area (55.07 m^2^ g^−1^) due to its nanosized particles and porous aggregates. After loading NiSe_2_ nanooctahedra onto P25 (TONS-15), the surface area decreases to 31.16 m^2^ g^−1^. Interestingly, unlike previously reported NiSe_2_/TiO_2_ systems, where performance enhancement was attributed to increased pore volume and improved dispersion,^43^ the present work shows a decrease in surface area and pore volume after loading NiSe_2_ nanooctahedra. This reduction can be attributed to the partial coverage of TiO_2_ nanoparticles by the relatively larger NiSe_2_ octahedral structures. Despite this decrease, a significant enhancement in photocatalytic activity is observed, indicating that surface area and porosity are not the dominant factors governing the activity in this system. Instead, the improved performance may be related to favorable charge transfer, enhanced electron separation, and the catalytic role of NiSe_2_ as an electron sink and active site for hydrogen evolution. This highlights the critical importance of electronic interactions over textural properties in determining photocatalytic efficiency.

### 3.1 Photocatalytic hydrogen production

**Fig. 6:**
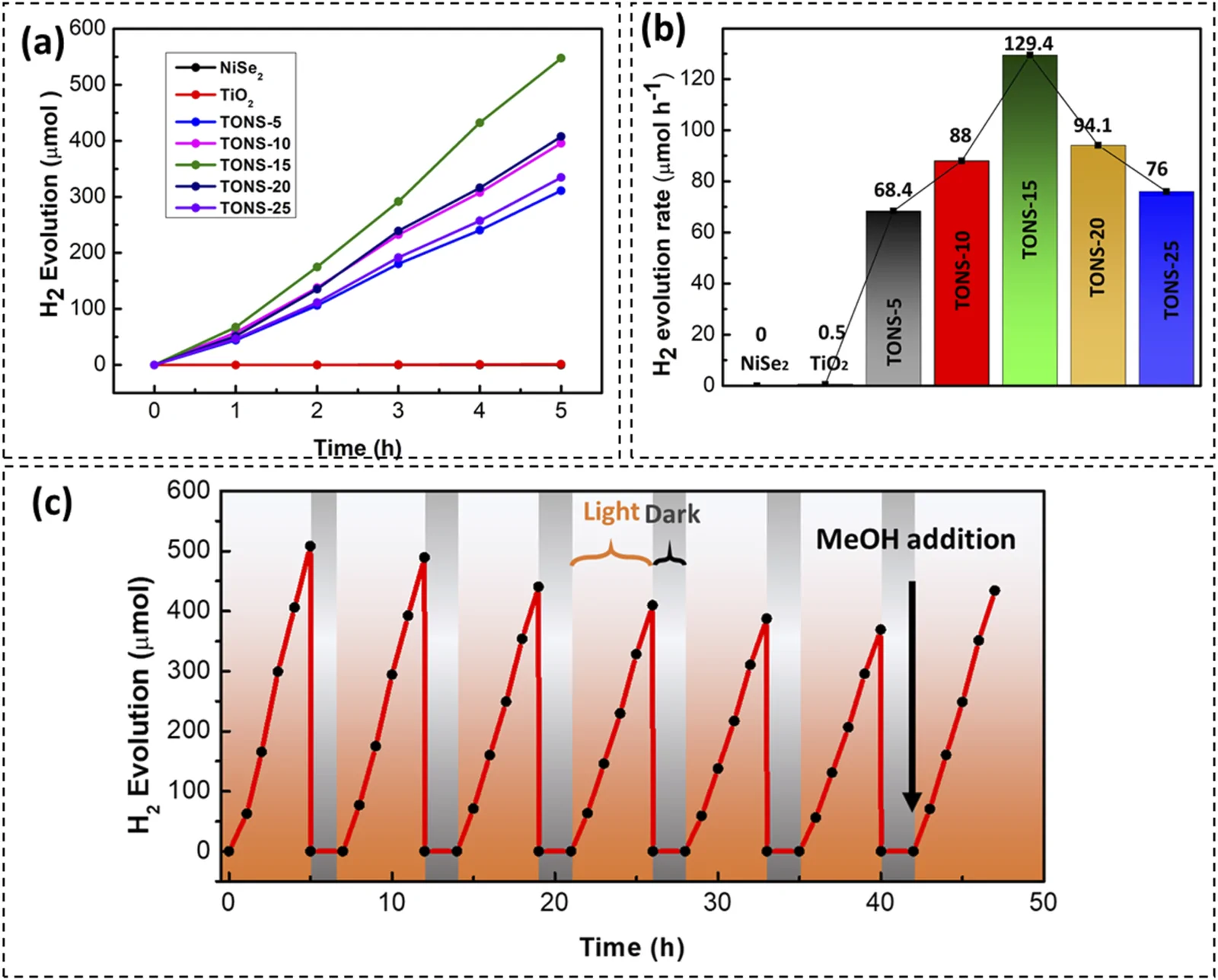
(a) Photocatalytic hydrogen evolution performance and (b) hydrogen evolution rate of NiSe_2_/TiO_2_ composites with varying NiSe_2_ loading for 5 hours. (c) Stability test of TONS-15 photocatalyst under cyclic photocatalytic hydrogen production for a total of 47 hours.

The photocatalytic hydrogen evolution performance of pure NiSe_2_ nanooctahedra, TiO_2_, and NiSe_2_-loaded TiO_2_ with 5, 10, 15, 20, and 25 wt% loadings was carried out. As can be seen from Fig. 6a, NiSe_2_ shows no hydrogen production over 5 h of illumination due to its metallic nature, while the hydrogen evolution efficiency of pristine TiO_2_ generates a low amount of hydrogen of about 0.5 µmol h^−1^ because of fast charge recombination. The hydrogen evolution rate increases with increasing NiSe_2_ loading up to 15 wt%, reaching 68.4, 88.0, and 129.4 µmol h^−1^ for TONS-5, TONS-10, and TONS-15, respectively. Beyond this optimum, the activity decreases to 94.1 and 76.0 µmol h^−1^ for TONS-20 and TONS-25, respectively (Fig. 6b). The superior performance of TONS-15 corresponds to an approximately 258-fold enhancement compared to pristine TiO_2_. This enhancement is attributed to the role of NiSe_2_ in promoting efficient charge separation and facilitating interfacial electron transfer, as supported by the XPS, PL, photocurrent, and EIS results. The remarkable decrease after reaching the maximum can be attributed to the surface covering of TiO_2_ and the light-shielding effect when loading with a high amount of the accumulation of relatively large NiSe_2_ nanooctahedra on the TiO_2_ surface. Compared with previously reported Ni-based cocatalysts, these results show competitive hydrogen evolution. NiS-loaded TiO_2_ was reported to produce 18.6 µmol h^−1^ under 365-nm UV LED,^44^ while Lu *et al.*^43^ reported a hydrogen evolution rate of 32 µmol h^−1^ for NiSe_2_ nanoparticles-loaded TiO_2_ under a 365 nm light source. Table S3 presents the hydrogen evolution rates of some reported noble and non-noble metal cocatalysts from the literature. Although noble metals might exhibit relatively higher hydrogen evolution rates, the present NiSe_2_/TiO_2_ achieves competitive activity while avoiding the use of scarce and expensive noble metals, demonstrating that NiSe_2_ nanooctahedra are promising cocatalysts for hydrogen production. To investigate the stability of the mixture, light/dark periods were switched for a total of 47 hours (5 hours of light followed by 2 hours of dark). As shown in Fig. 6c, there is a slight decrease in hydrogen evolution. This phenomenon corresponds to the observed reduction of the water/methanol solution volume after cycle 6, where the volume reduced from 100 mL to about 91 mL. Since methanol acts as a hole scavenger, its removal limits the efficient consumption of photogenerated holes, resulting in increased charge recombination and consequently lower hydrogen production. To further confirm this assumption, directly after cycle 6, we introduced 5 mL of methanol, and the hydrogen production increased again, indicating that one of the factors that may contribute to this decrease in hydrogen is methanol consumption and not catalyst degradation. To check the XRD after 47 hours of reaction, the resulting suspension was washed, and the powder was collected by centrifuging, followed by drying. As shown in Fig. S10, there is no change in the XRD peaks positions, demonstrating its structural stability for a long reaction. Post-reaction XPS analysis was conducted to further investigate the stability of the used catalyst. The results confirmed that the characteristic features of TONS-15 were retained after the stability test, indicating that the NiSe_2_ is largely preserved during the reaction. The Ni 2p fitting reveals that no additional Ni chemical-state components were detected (Fig. S11a). Similarly, the Selinide phase remains detectable after reaction, as shown in Fig. S11b. However, a decrease in Se 3d intensity was observed, while the oxidized selenium species (SeO_2_/SeO_*x*_) were found to be unchanged after the stability test. These results indicate that the decrease in the Se signal is not associated with Se oxidation. Instead, as shown in Table S4, ICP-OES analysis of the post-reaction solution detected low Ni and Se concentrations, while Ti was below the detection limit, confirming partial surface leaching of the NiSe_2_ cocatalyst during photocatalysis. A marked increase in the C 1s signal and a lower apparent atomic percentage of Ti, Ni, and Se were observed from post-XPS analysis, suggesting the formation of a carbonaceous overlayer during methanol conversion in the photocatalytic reaction. In fact, carbonaceous surface species have been reported to form during methanol decomposition through C–O bond scission of methanol-derived intermediates, where they can accumulate on the catalyst surface and partially block active sites.^45^ The combined XRD, XPS, and ICP-OES results indicate that the catalyst maintains its crystalline structure and preserves the majority of the NiSe_2_ cocatalyst after prolonged reaction, while undergoing partial surface NiSe_2_ leaching together with carbonaceous surface coverage, rather than bulk structural degradation.

**Fig. 7:**
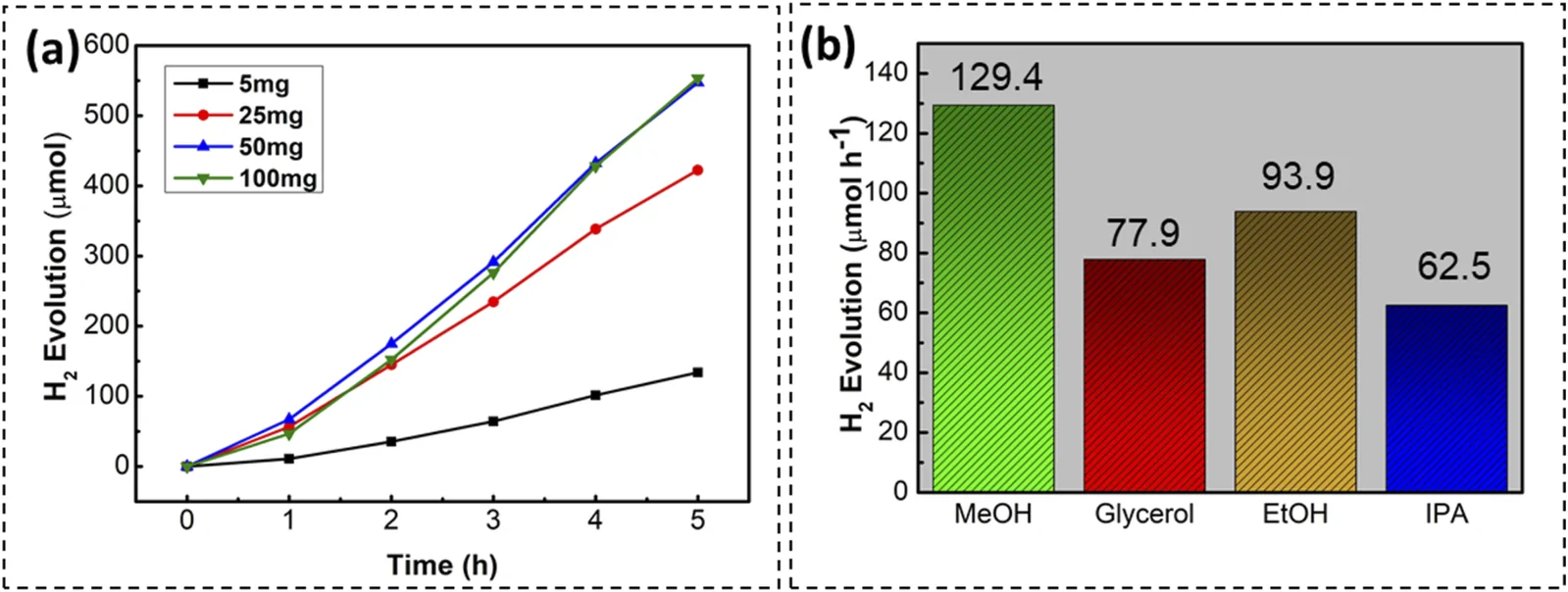
(a) Effect of catalyst amount (5–100 mg) on hydrogen evolution over time. (b) Comparison of hydrogen evolution using different sacrificial agents (MeOH, glycerol, EtOH, IPA).

The hydrogen rate using 50 mg was higher compared to pristine TiO_2_. To check the effect of the amount of the catalysts, different amounts, including 5, 25, 50, and 100 mg, were investigated, as shown in Fig. 7a. The total hydrogen evolution increases with increasing catalyst amount from 5 to 50 mg, indicating that a large number of photoactive sites are available, leading to enhanced light conversion capability. For loadings of 50 to 100 mg the hydrogen production remains nearly constant, indicating that the reactor reaches an optical saturation regime around 50 mg, where photon availability and light penetration become limiting rather than the catalyst amount.

To further understand the effect of catalyst loading, UV-Vis transmission spectra were recorded for different catalyst amounts using a cuvette with an optical path length of 3 mm (Fig. S12). At higher catalyst loading, the transmitted light intensity decreases across the entire spectral range, indicating that the suspension becomes more opaque. Specifically, at 365 nm, the transmission decreases from approximately 78% for 5 mg to 2.6% for 50 mg. In fact, to benefit from the whole catalyst's activity in a system, the light should cover the whole reactor volume, and what is observed is that at higher loading, strong light attenuation limits photon penetration into the reaction medium. As a result, a significant fraction of the catalyst particles remains insufficiently illuminated. Consequently, increasing the catalyst loading beyond a certain level (>50 mg) does not proportionally enhance hydrogen evolution. In contrast, lower to moderate catalyst loadings favor a more balanced photon distribution throughout the suspension.

The role of a sacrificial agent is to act as a hole scavenger, thereby suppressing charge recombination and enhancing hydrogen production. But the optimum concentration differs from one catalyst to another. To further investigate the methanol concentration effect, methanol/water mixtures containing 10, 25, 50, 75, and 99% were used, and the results are shown in Fig. S13. The highest hydrogen evolution is found at 75, however, after 75%, there is not too much change, indicating that the optimum is at 75%. These findings are in contrast to many reported findings, which state that at near 100%, there is a drop in photocatalytic activity as the excess of methanol molecules gets adsorbed on the catalyst's surface.^46^ Our results show nearly constant activity from 75 to 99% of methanol concentrations, indicating that no significant surface blocking, mass transfer limitation, or proton depletion occurs. Instead, above 75%, the system reaches a saturation regime, in which the rate is governed by photogenerated charge carrier flux rather than sacrificial agent concentration. Since reactive species needed for methanol oxidation are constant and only depend on light intensity, irradiation time, and catalyst amount.

The influence of the sacrificial agent on photocatalytic hydrogen evolution was investigated using methanol, ethanol, isopropanol, and glycerol under identical conditions (Fig. 7b). Among these, methanol exhibited the highest hydrogen evolution performance, which can be attributed to its simple molecular structure and rapid oxidation kinetics. During irradiation, methanol is efficiently oxidized by photogenerated holes, suppressing charge recombination and enhancing hydrogen production. Gas chromatography analysis confirmed CO_2_ as the main detectable gaseous oxidation product under the applied conditions, suggesting extensive methanol oxidation. In contrast, glycerol (C_3_H_8_O_3_), a by-product of biodiesel production, exhibits more complex photocatalytic behavior due to its polyfunctional structure. Gas chromatography (GC) analysis revealed the formation of H_2_ along with both CO and CO_2_, indicating that glycerol reforming proceeds *via* the coexistence of partial oxidation and complete mineralization pathways. The presence of CO suggests incomplete oxidation and decarbonylation reactions, whereas CO_2_ formation reflects deeper oxidative processes. Post-reaction ^1^H NMR analyses were collected after 60 and 300 min of reaction, corresponding to approximately 61.6 and 457 µmol of produced H_2_, respectively. The results did not reveal major detectable liquid-phase intermediates within the sensitivity limits of the measurement (Fig. S14), indicating that any intermediate species are either present at low concentration, rapidly converted, or adsorbed on the catalyst surface. These findings demonstrate that, unlike methanol, glycerol undergoes more complex reforming pathways involving transient surface-bound intermediates and multi-step electron transfer processes.

From the obtained experimental results, a possible photocatalytic mechanism for the NiSe_2_/TiO_2_ heterostructure is proposed. When TiO_2_ is subjected to light, the electrons and holes are generated, and the electrons are excited from the valence band to the conduction band, leaving behind holes. Due to the favorable interfacial contact and the metallic conductivity of NiSe_2_, the photogenerated electrons are expected to be transferred from the conduction band of TiO_2_ to NiSe_2_. The accumulated electrons on NiSe_2_ are proposed to participate in proton reduction, while the residual positive charge holes in the VB of TiO_2_ interacted with the sacrificial agent, which suppresses electron–hole recombination and enhances charge-separation efficiency, as supported by the PL quenching, photocurrent and EIS measurements. However, excessive loading of NiSe_2_ can lead to light-shielding effects and blockage of active sites, which explains the observed decline in activity at higher loadings.

## 4 Conclusion

This work presents an effective approach to improve the photocatalytic performance of TiO_2_ for the production of hydrogen, highlighting the role of morphology-controlled NiSe_2_ nanooctahedra co-catalysts as a promising solution. NiSe_2_ nanooctahedra were first synthesized by a simple hydrothermal method and then used as an efficient non-noble co-catalyst. The optimum NiSe_2_ loading was found around 15 wt%, leading to 129.4 µmol h^−1^ and an apparent quantum efficiency of ≈4% at 365 nm, which represents a 258-fold increase compared to pristine TiO_2_. This enhancement is attributed to the reduced radiative recombination and improved charge separation, as supported by XPS peak shifts, PL, and EIS analysis. The results emphasize the crucial significance of the catalyst loading, catalyst amount, and sacrificial agent type, revealing that photocatalytic efficiency is governed not only by the available catalyst surface but also by light penetration within the reaction system. Overall, this study demonstrates that morphology-controlled NiSe_2_ nanooctahedra provide a cost-effective, stable, and efficient strategy for enhancing photocatalytic hydrogen evolution.

## Conflicts of interest

The authors declare that they have no known competing financial interests or personal relationships that could have appeared to influence the work reported in this paper.

## Supplementary Material

RA-016-D6RA04767J-s001

## Data Availability

The data supporting the findings of this study are available within the article and its supplementary information (SI). Supplementary information: details of the photocatalytic and photoelectrochemical setups, AQE determination and photon flux measurements, Rietveld refinement, EDX, XPS, UV-Vis and BET characterization, catalyst stability analysis, UV-Vis suspension measurements, the effect of methanol concentration, ^1^H NMR spectra, ICP-OES analysis, and a comparison of the photocatalytic hydrogen evolution performance with previously reported TiO_2_-based photocatalysts. See DOI: https://doi.org/10.1039/d6ra04767j.
