# Supplementary material for: Boosting photocatalytic hydrogen evolution through efficient charge separation in NiSe2 nanooctahedra-TiO2 heterostructures
Source: RSC Adv. 2026 Aug 11;16(40):46613–22. doi: 10.1039/d6ra04767j (PMC13458687; doi:10.1039/d6ra04767j)
Supplement: Supplementary file 1 [file RA-016-D6RA04767J-s001.pdf]

Supplementary Information (SI) for RSC Advances.
This journal is © The Royal Society of Chemistry 2026

Boosting Photocatalytic Hydrogen Evolution through Efficient Charge Separation in $NiSe_2$ Nanooctahedra–TiO_2 Heterostructures

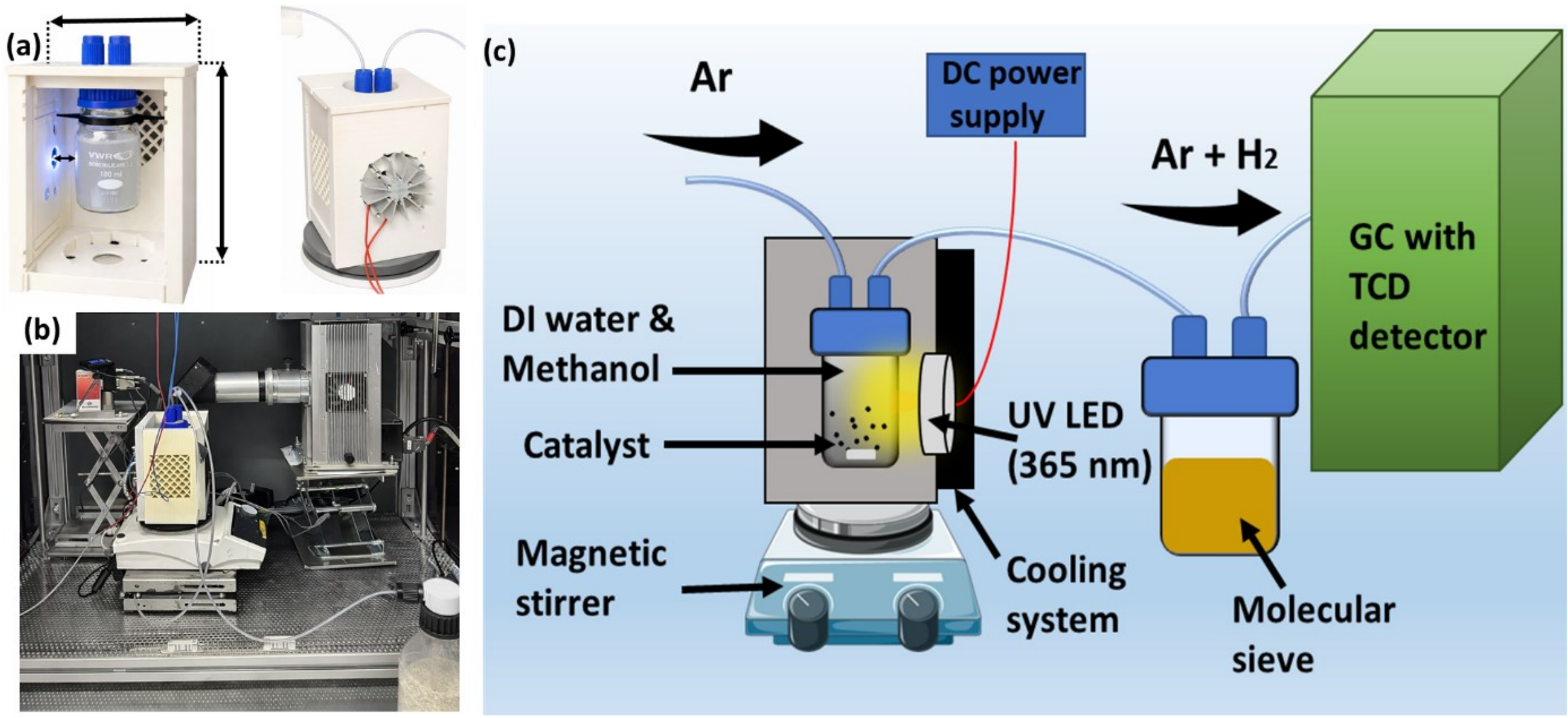

Fig. S1. (a) Compact UV LED photoreactor module showing front and side views with integrated cooling system. (b) Photograph of the experimental setup used for photocatalytic hydrogen production. (c) Schematic diagram of the photocatalytic reaction and gas analysis system. The reaction is carried out in a photoreactor containing DI water/methanol as a sacrificial agent and catalyst under UV LED (365 nm) irradiation. Argon (Ar) is used as a carrier gas. The evolved gas mixture passes through a moisture trap (molecular sieve) to remove water vapor before being analyzed by gas chromatography (GC) equipped with a thermal conductivity detector (TCD).

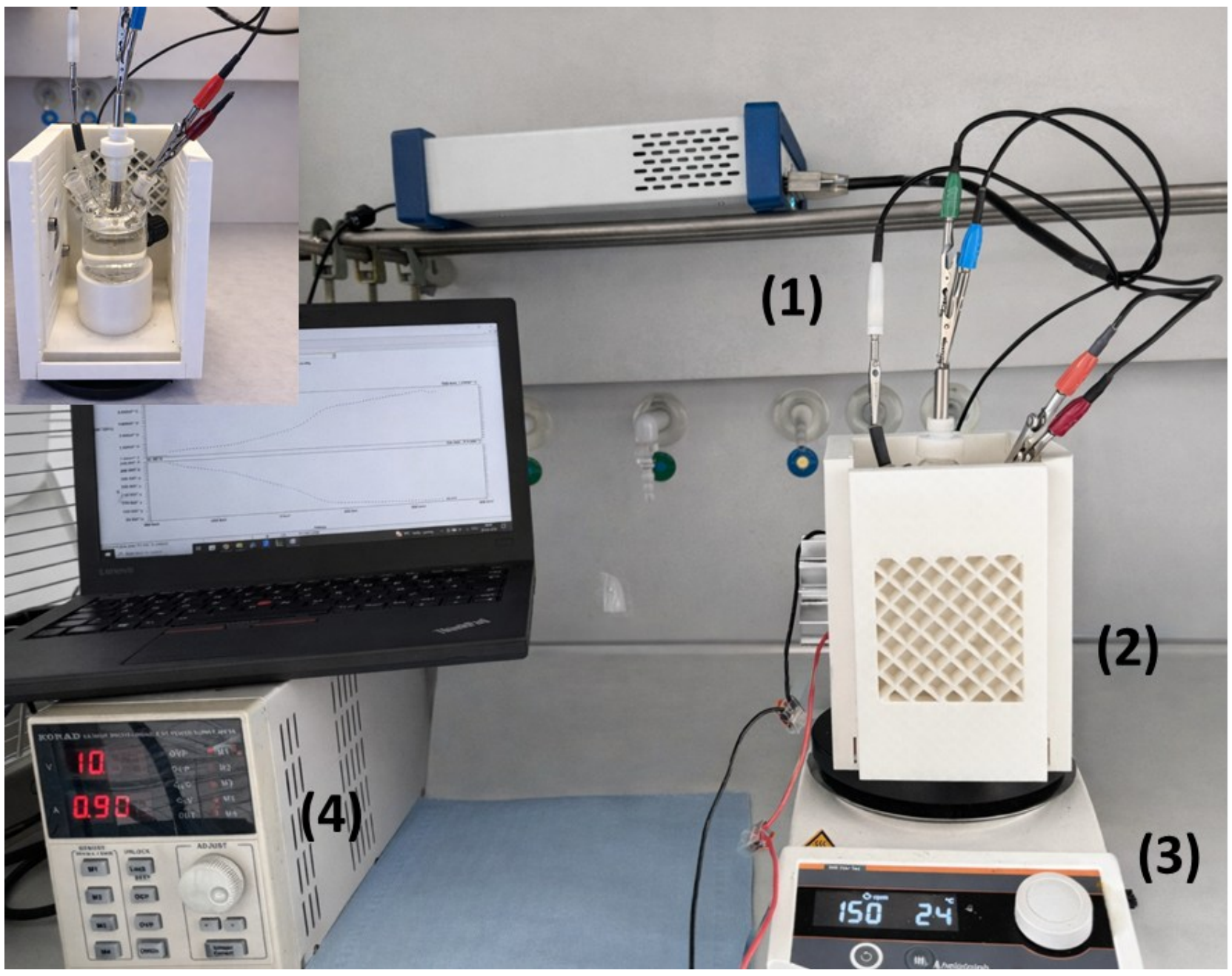

Fig. S2. Experimental setup for electrochemical measurements. The system consists of (1) Multiple electrodes (working, counter, and reference electrodes) connected with an electrochemical workstation, (2) a modular photoreactor chamber, (3) a magnetic stirrer/hotplate used for temperature control and mixing, and (4) a DC power supply providing controlled current. The inset shows a close-up of the reactor vessel containing the reaction medium and electrode configuration.

AQE calculation:

Determination of Photon Flux by Ferrioxalate Actinometry

The photon flux of the LED light source (λ = 365 nm) was determined using a potassium ferrioxalate actinometer, following the procedure reported by Ziegenbalg et al.[1–3]. First, a ferrioxalate solution was prepared in a red-light room and transferred into a 100 mL photoreactor. The solution was irradiated under identical conditions as the photocatalytic experiments (same reactor geometry, LED position, and current = 700 mA). 0.5 mL of the solution at 0, 30, 90, 120, 240, and 360s was transferred into 19 mL of 0.05 M H_2SO_4. Subsequently, 5 mL of this solution was mixed with 15 mL of 1,10-phenanthroline developer solution. The absorbance of the resulting $[Fe(phen)_3]^{2+}$ complex was measured at 510 nm.

The actinometer conversion and Fe^{2+} concentration were calculated using equations 1 and 2, respectively (Fig. S3a):

$$X = \frac{n_{Fe^{2+}}}{\eta_0} \quad \text{(Eq. 1)}$$

$$c = \frac{A_{510}}{\varepsilon l} \quad \text{(Eq. 2)}$$

$n_{Fe^{2+}}$: The amount of Fe^{2+} generated.

n_0: Initial amount of ferrioxalate in the reactor.

A_{510}: The measured absorbance.

ε: 1.1×104 $L \cdot mol^{-1} \cdot cm^{-1}$ and l: 1cm

The number of absorbed photons and the resulting absorbed photon flux (q_{abs}) was calculated using equations 3 and 4, respectively:

$$n_{photons,abs} = \frac{n_{Fe^{2+}}}{\Phi} \quad \text{(Eq. 3)}$$

$$q_{abs} = \frac{n_{photons,abs}}{t} \quad \text{(Eq. 4)}$$

Φ : the quantum yield of ferrioxalate at the irradiation wavelength (1.25).

The photon flux remained approximately constant over the investigated time range, with an average value of q_{abs} =1800 nmol s^{-1} (Fig. S3b).

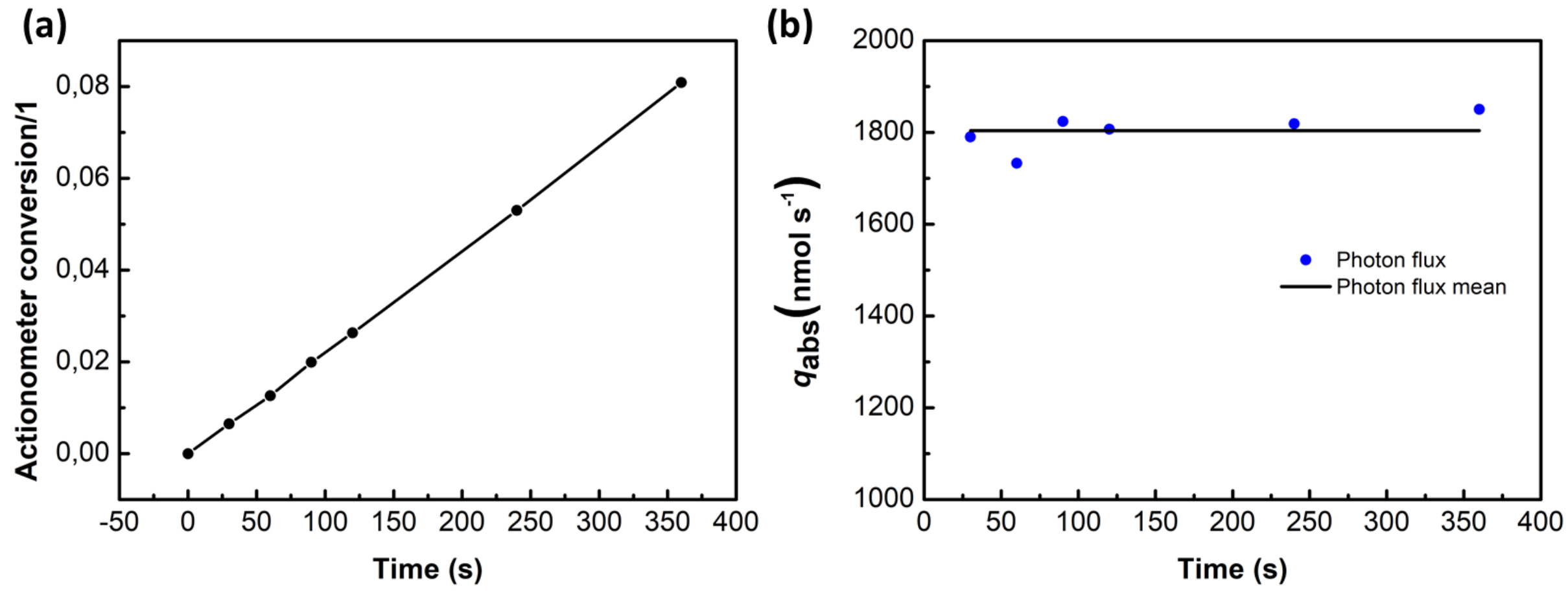

Fig. S3. (a) Actinometer conversion and (b) calculated photon flux over time for 365 nm LED at 0.7 A within the modular photoreactor under constant stirring

Determination of AQE

The apparent quantum efficiency (AQE) for hydrogen evolution was calculated using:

$$AQE(\%) = \frac{2r_{H_2}}{q_{abs}} \times 100 \qquad \text{(Eq. 5)}$$

r_{H_2}:The hydrogen measured (µmol h^{-1}). The factor of 2 accounts for the two electrons required to produce one molecule of H_2.

q_{abs}: 1800 nmol s^{-1}

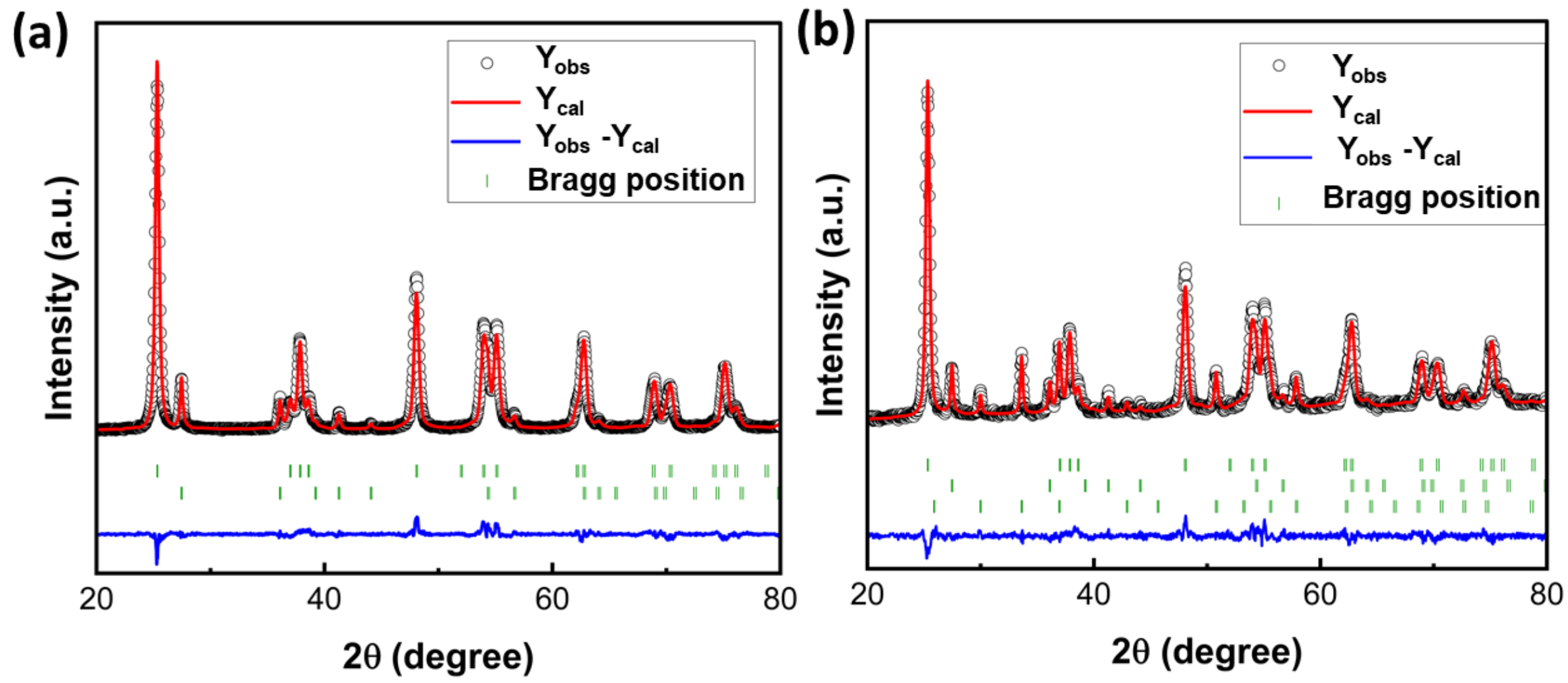

Fig. S4. Rietveld refinement of (a) TiO_2 and (b) TONS-15.

Table S1: Lattice parameters of TiO_2 (anatase and rutile phases) and $NiSe_2$ (cubic phase) before and after integration.

Phases \ Compounds	TiO_2 (P25) (Å)	$NiSe_2$ (Å)	TONS-15 (Å)
TiO_2 (Anatase phase)	a=b= 3.78575<br>c= 9.50748		a=b=3.78588<br>c= 9.50409
TiO_2 (Rutile phase)	a=b= 4.59494<br>c=2.96034		a=b= 4.59320<br>c= 2.95991
$NiSe_2$ (Cubic phase)		a=b=c= 5.96072	a=b=c= 5.96141

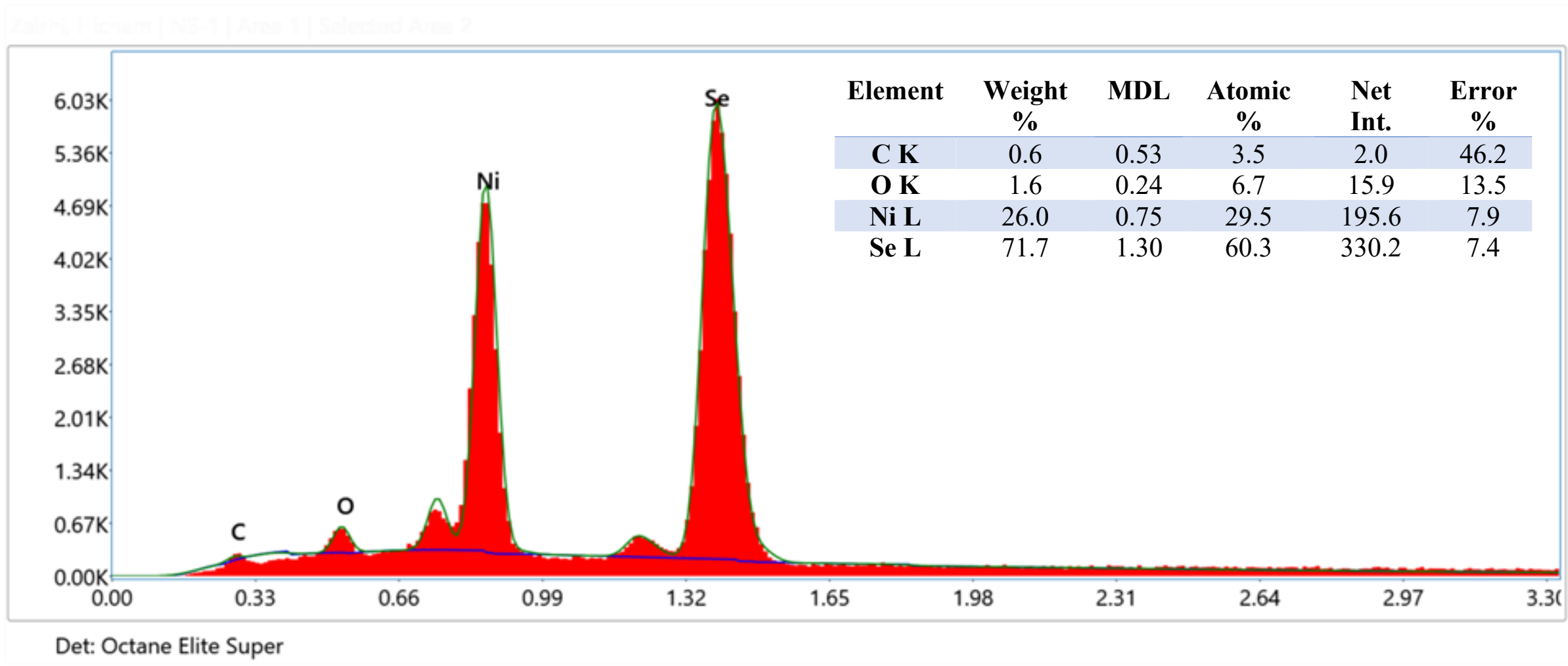

Fig. S5. EDX analysis of $NiSe_2$

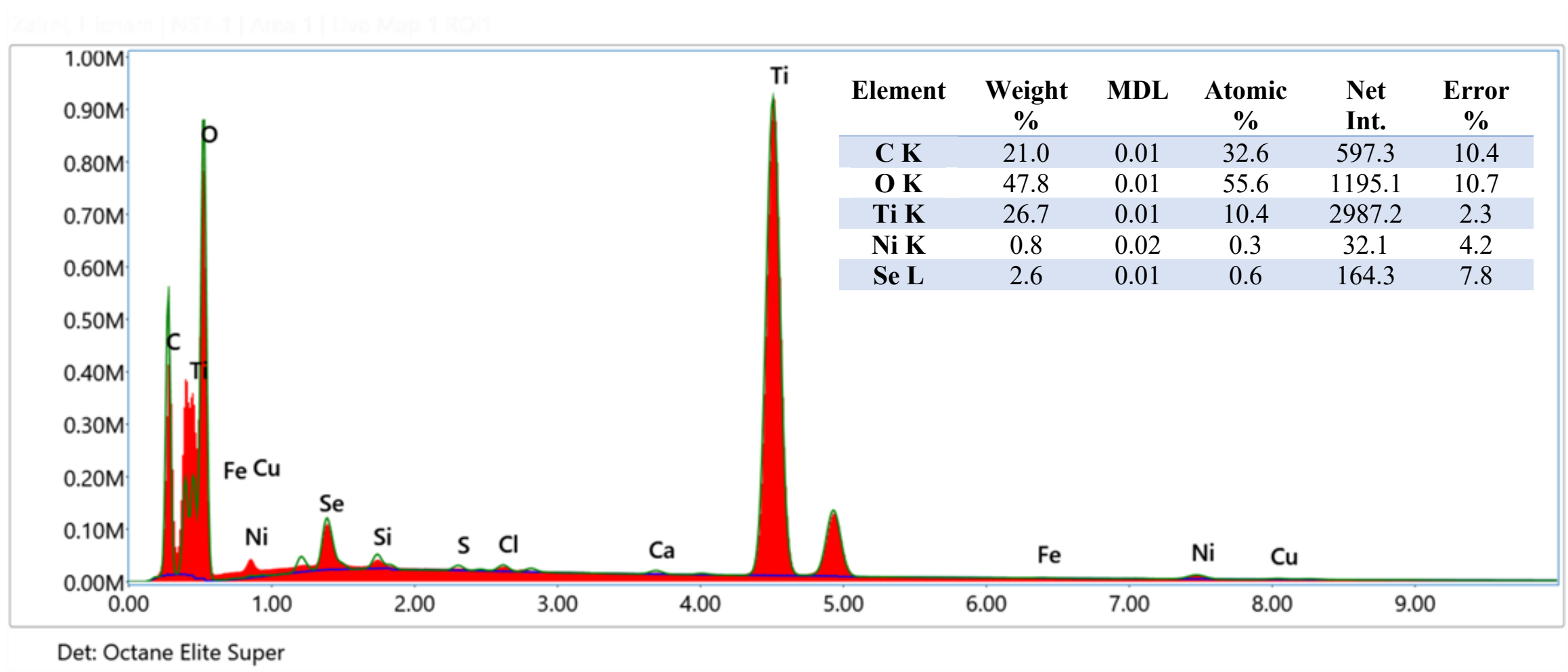

Fig. S6. EDX analysis of TONS-15.

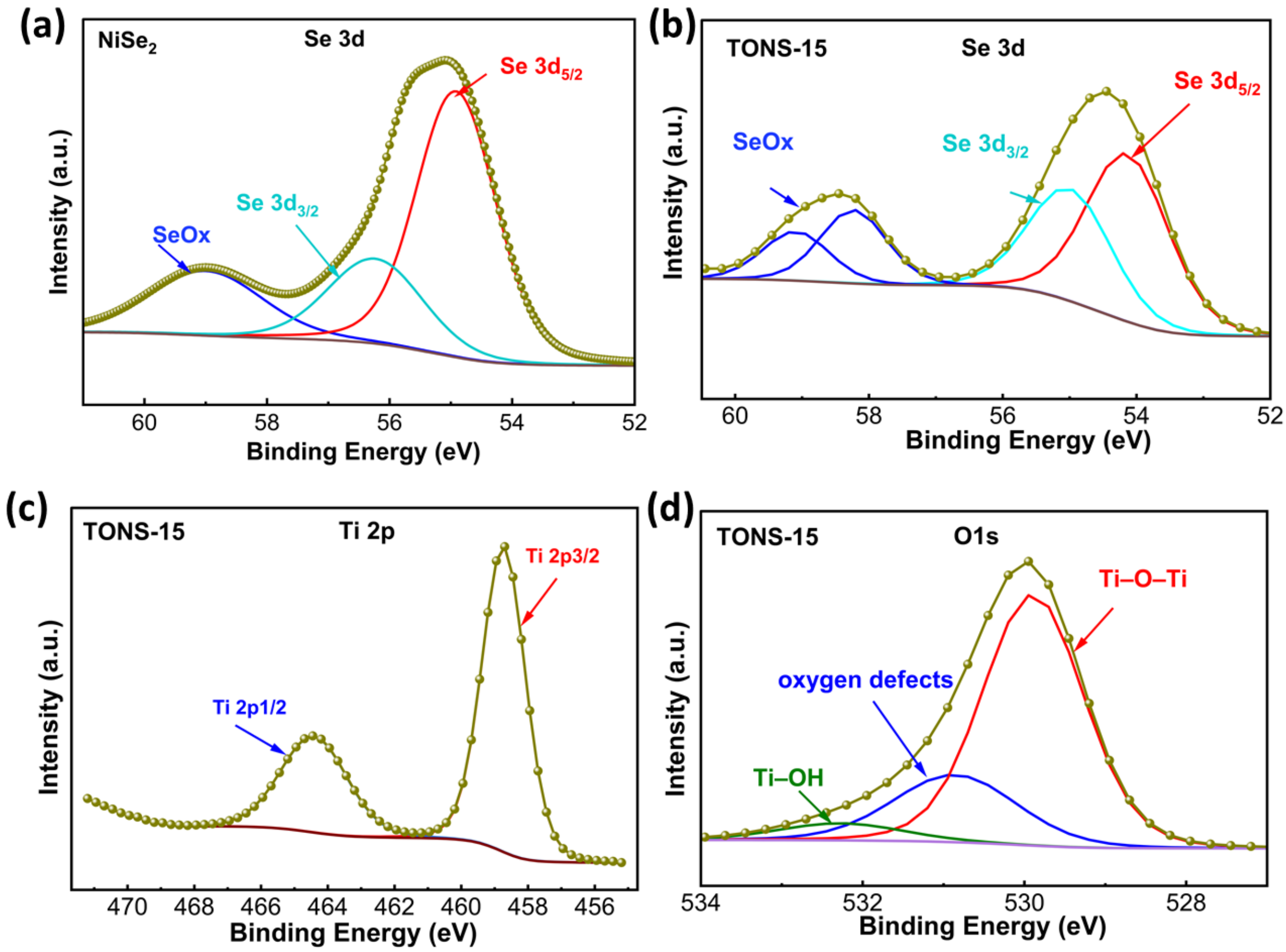

Fig. S7. High resolution spectra of (a) Se 3d for $NiSe_2$ sample and (b) Se 3d, (c) Ti 2p, (d) O 1s for TONS-15 sample.

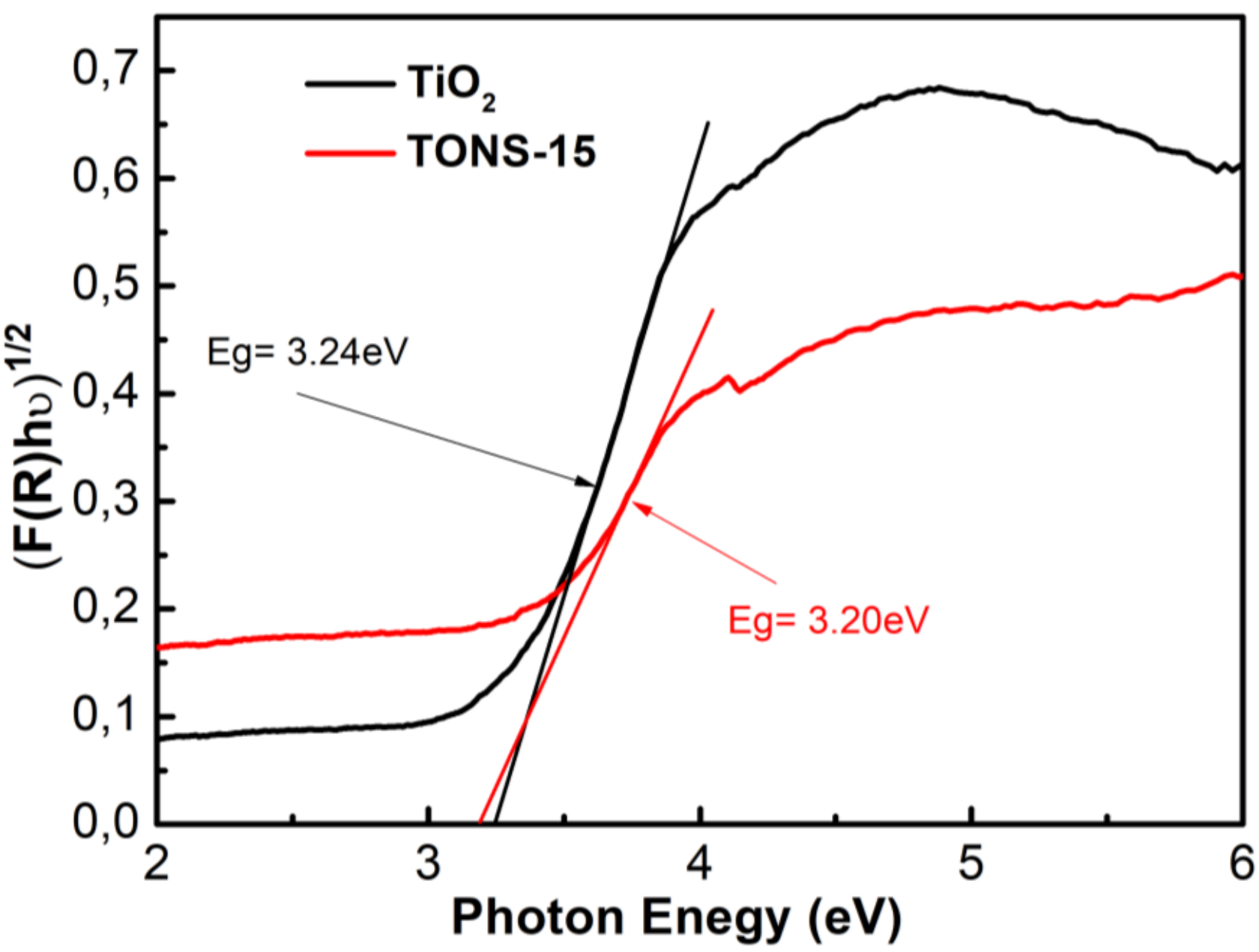

Fig. S8. Kubelka–Munk transformed Tauc plots for pristine TiO_2 and TONS-15, used to estimate the indirect optical band-gap energies

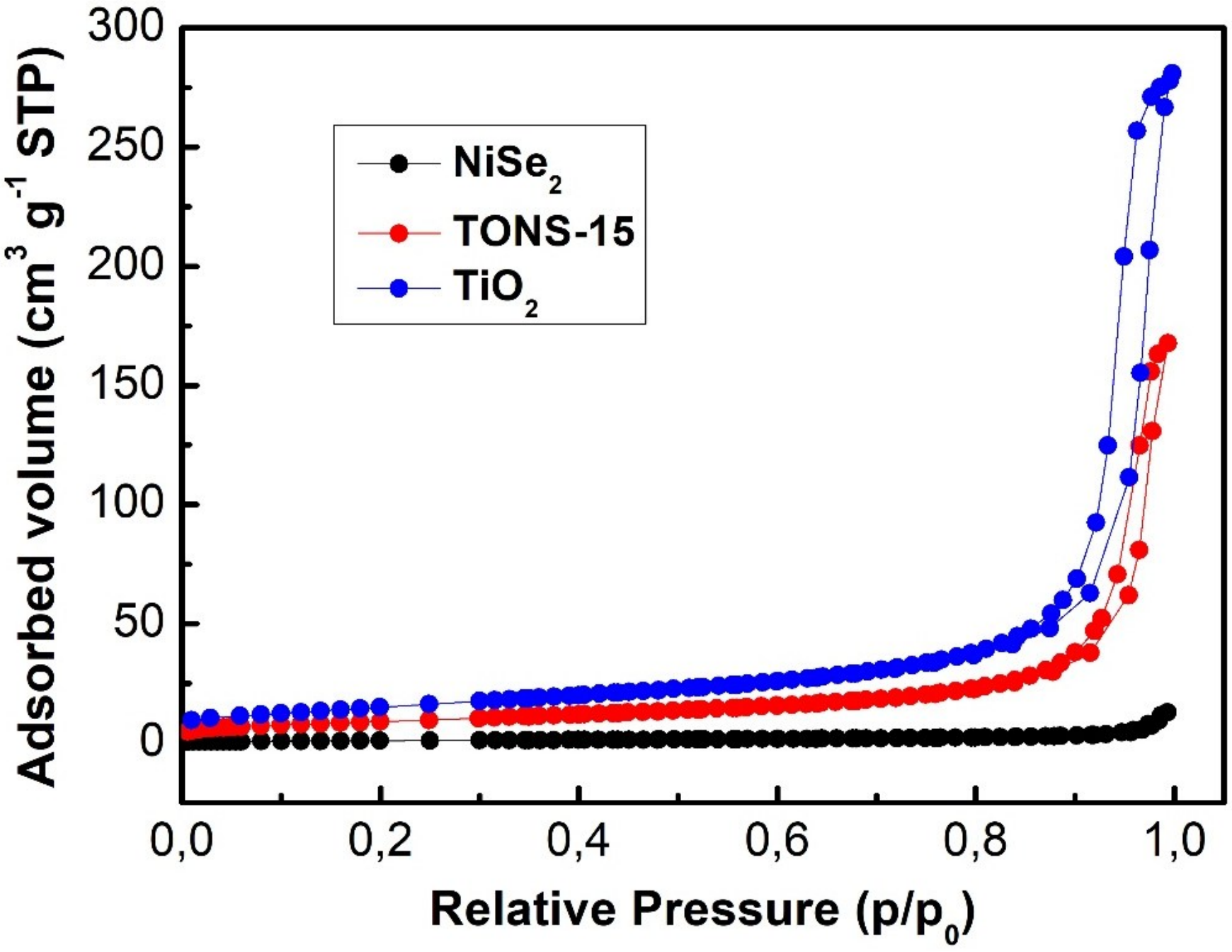

Fig. S9. N_2 adsorption–desorption isotherms of $NiSe_2$, TONS-15, and TiO_2 at 77 K showing mesoporosity in TiO_2 and reduced porosity upon $NiSe_2$ loading.

Table S2. Summary of key BET parameters of TiO_2, TONS-15, and $NiSe_2$ samples.

**Sample**	**Surface Area (m^2/g)**	**Pore Volume (cm^3/g)**	**Porosity**
TiO_2 (P25)	55.07	0.0226	Mesoporous
$NiSe_2$	3.63	0.0015	Non-porous
TONS-15	31.16	0.0135	Reduced mesoporosity

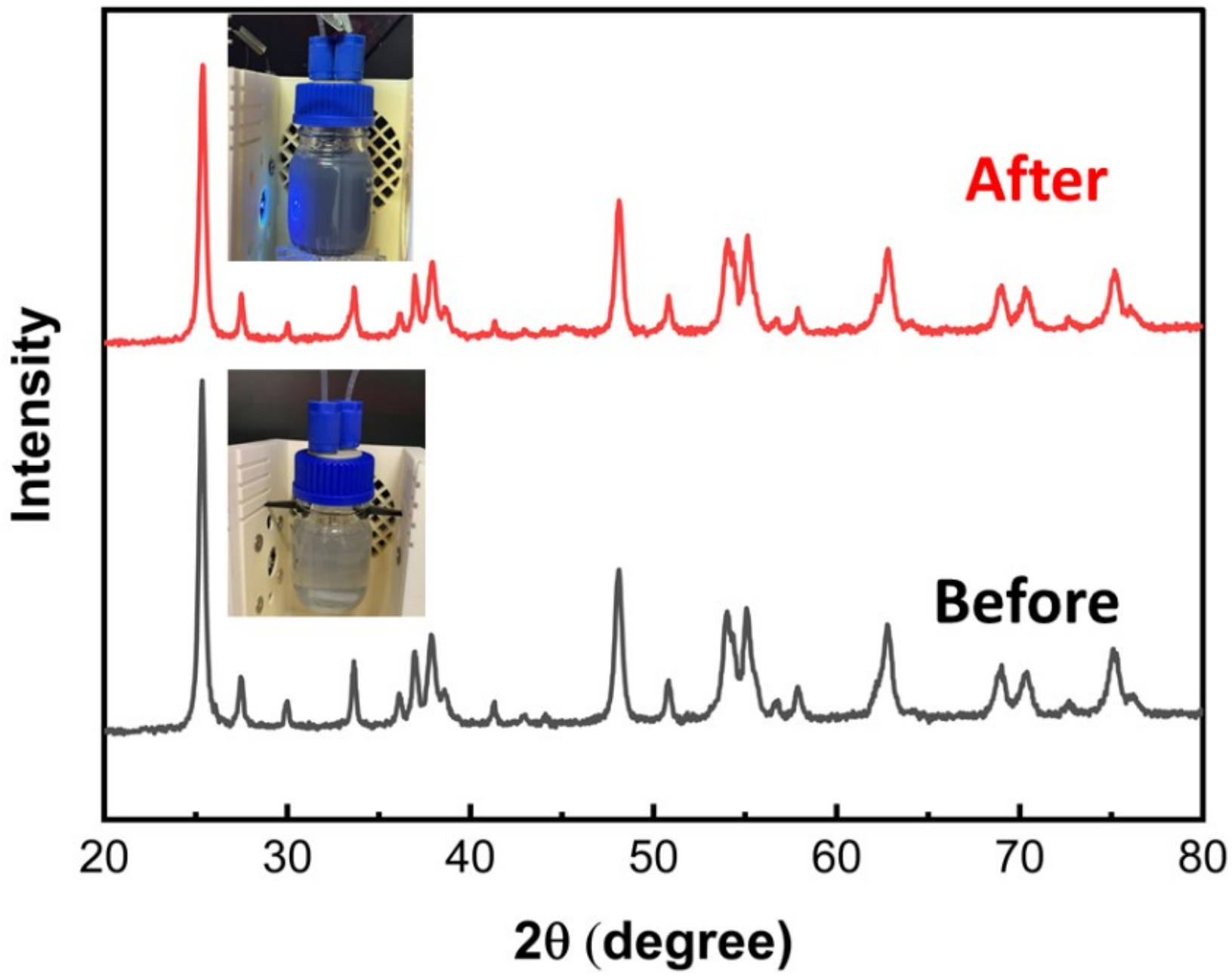

Fig. S10. XRD patterns of the TONS-15 sample before and after 47 hours of reaction.

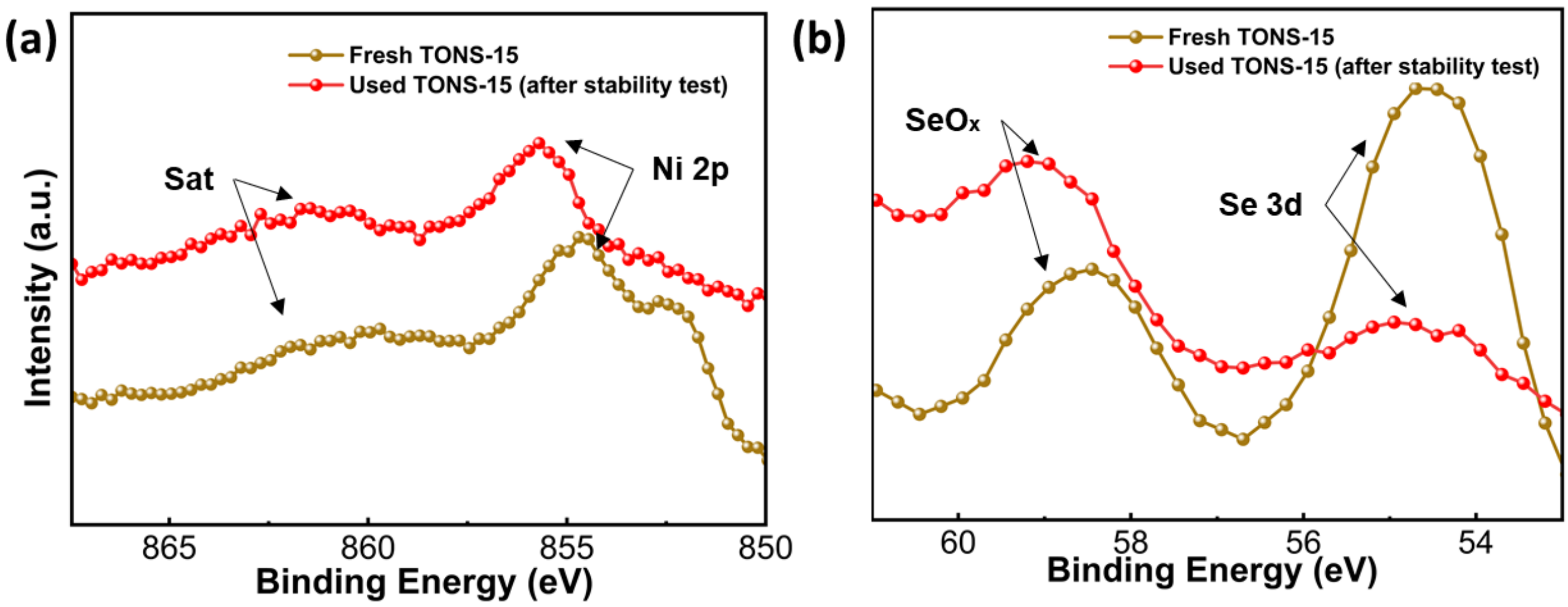

Fig. S11. High-resolution XPS spectra of (a) Ni 2p and (b) Se 3d for fresh and used TONS-15 after photocatalytic stability test

Table S3. Comparison of photocatalytic H_2 evolution performance of Ni-based TiO_2 photocatalysts reported in the literature.

Composite photocatalyst	Pristine activity (μmol h^{-1} g^{-1})	Composite activity (μmol h^{-1} g^{-1})	Enhancement factor	Light source	Reference
0.2 wt% Pt/TiO_2	-	3400	-	Two UV lamps	[4]
1 wt% Pt/TiO_2	-	1638	-	Solar simulator	[5]

0.3 wt% AuPd/TiO_2	-	526	-	Xe lamp	[6]
Rh/TiO_2	252.6	4900	-	UV-pen ray Hg lamp	[7]
NiSe/TiO_2	-	1108	×11	Xe lamp	[8]
NiS/TiO_2	-	698	×30	300 W Xe lamp	[9]
Ag/Ni-TiO_2	37.28	2933.9	×78.9	365 nm LED	[10]
$Ni(HCO3)_2/TiO_2$	98.6	1798.0	×18	300 W Xe lamp	[11]
$NiSe_2$ Nanooctahedra/ TiO_2	10	2588	×258	365 nm LED (0.7 A)	This work

Table S4. ICP-OES analysis of the post-reaction solution after photocatalytic stability test of TONS-15.

Element	Mass Concentration (mg/L)	RSD (%)
Ni	0.5195	0.625
Se	1.516	07.294
Ti	Not detected	-

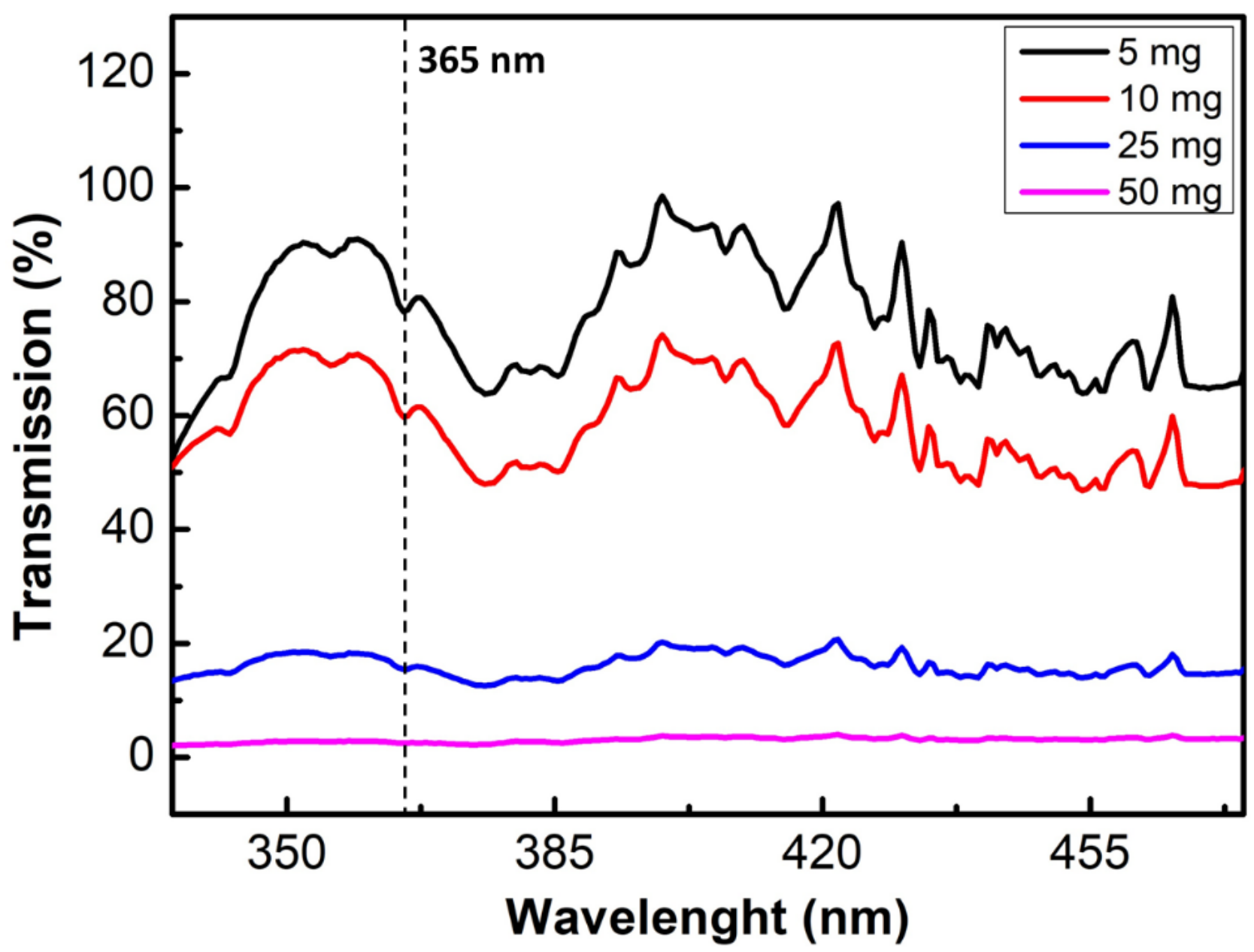

Fig. S12. UV-Vis spectra of suspension for 0, 5, 10, 25, 50 mg of catalysts in methanol/water solution using a 3mm optical path length cuvette.

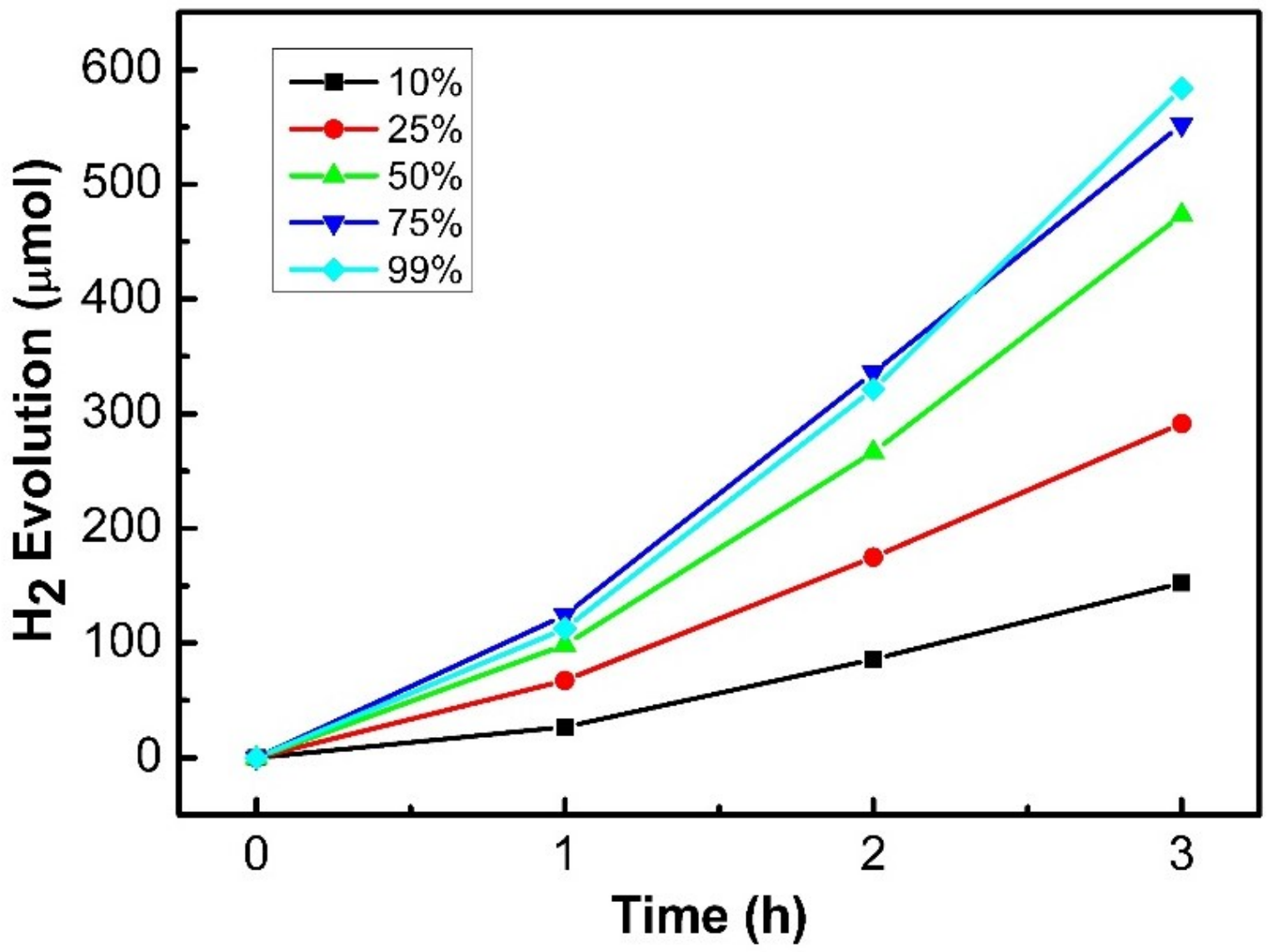

Fig. S13. Effect of methanol concentration (10–99%) on hydrogen evolution performance.

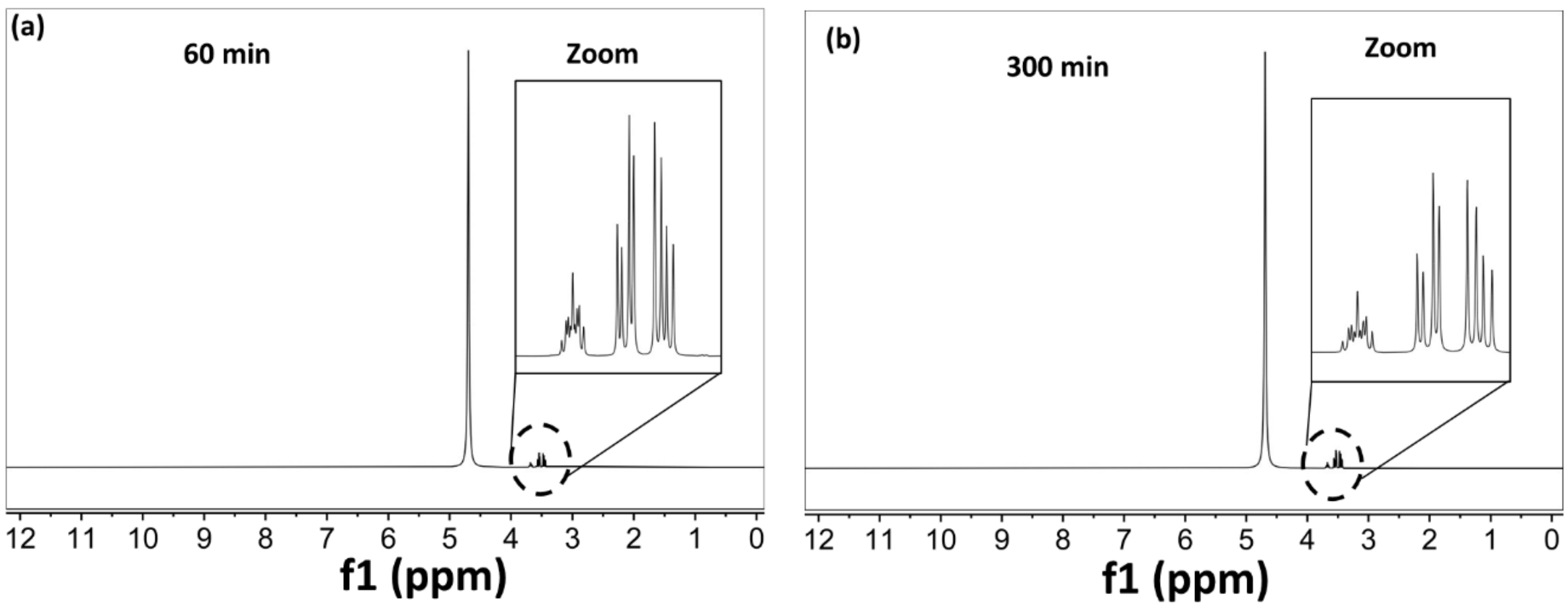

Fig. S14. ^{1}H NMR spectra of the reaction solution after (a) 60 min and (b) 300 min of photocatalytic glycerol reforming.

1 D. Kowalczyk, P. Li, A. Abbas, J. Eichhorn, P. Buday, M. Heiland, A. Pannwitz, F. H. Schacher, W. Weigand, C. Streb and D. Ziegenbalg, *ChemPhotoChem*, 2022, **6**, e202200044.

2 B. Wriedt and D. Ziegenbalg, *ChemPhotoChem*, 2021, **5**, 947–956.

3 B. Wriedt and D. Ziegenbalg, *J. Flow Chem.*, 2020, **10**, 295–306.

4 B.-S. Huang, F.-Y. Chang and M.-Y. Wey, *Int. J. Hydrog. Energy*, 2010, **35**, 7699–7705.

5 K. Shu, C. Chuaicham and K. Sasaki, *Chem. Eng. J.*, 2023, **477**, 146994.

6 Y. Xin, L. Wu, L. Ge, C. Han, Y. Li and S. Fang, *J. Mater. Chem. A*, 2015, **3**, 8659–8666.

7 R. Camposeco, M. Hinojosa-Reyes and R. Zanella, *Int. J. Hydrog. Energy*, 2021, **46**, 26074–26086.

8 H. Gong, Q. Liu and C. Huang, *Int. J. Hydrog. Energy*, 2019, **44**, 4821–4831.

9 L. Zhang, B. Tian, F. Chen and J. Zhang, *Int. J. Hydrog. Energy*, 2012, **37**, 17060–17067.

10 D. Gao, W. Liu, Y. Xu, P. Wang, J. Fan and H. Yu, *Appl. Catal. B Environ.*, 2020, **260**, 118190.

11 Y. Wei, G. Cheng, J. Xiong, F. Xu and R. Chen, *ACS Sustain. Chem. Eng.*, 2017, **5**, 5027–5038.
